# Tethering of CHROMATOR and dCTCF proteins results in decompaction of condensed bands in the *Drosophila melanogaster* polytene chromosomes but does not affect their transcription and replication timing

**DOI:** 10.1371/journal.pone.0192634

**Published:** 2018-04-02

**Authors:** Galina V. Pokholkova, Sergei A. Demakov, Oleg V. Andreenkov, Natalia G. Andreenkova, Elena I. Volkova, Elena S. Belyaeva, Igor F. Zhimulev

**Affiliations:** 1 Institute of Molecular and Cellular Biology of the Siberian Branch of the Russian Academy of Sciences (IMCB RAS), Novosibirsk, Russia; 2 Novosibirsk State University (NSU), Novosibirsk, Russia; Universita degli Studi di Roma La Sapienza, ITALY

## Abstract

Instulator proteins are central to domain organization and gene regulation in the genome. We used ectopic tethering of CHROMATOR (CHRIZ/CHRO) and dCTCF to pre-defined regions of the genome to dissect the influence of these proteins on local chromatin organization, to analyze their interaction with other key chromatin proteins and to evaluate the effects on transcription and replication. Specifically, using UAS-GAL4DBD system, CHRO and dCTCF were artificially recruited into highly compacted polytene chromosome bands that share the features of silent chromatin type known as intercalary heterochromatin (IH). This led to local chromatin decondensation, formation of novel DHSes and recruitment of several “open chromatin” proteins. CHRO tethering resulted in the recruitment of CP190 and Z4 (PZG), whereas dCTCF tethering attracted CHRO, CP190, and Z4. Importantly, formation of a local stretch of open chromatin did not result in the reactivation of silent marker genes *yellow* and *mini*-*white* immediately adjacent to the targeting region (UAS), nor did RNA polII become recruited into this chromatin. The decompacted region retained late replicated, similarly to the wild-type untargeted region.

## Introduction

Domain organization of eukaryotic genomes can be broadly defined as partitioning of chromosomes into multiple chromatin blocks having autonomous chromatin state(s) and gene activity regulation. Spatial organization of eukaryotic genomes [1–5], particularly that of the *Drosophila* genome [6–9] has been comprehensively analyzed, which led to the proposal of a concept on the linear subdivision of genomes into discrete topologically associating domains (TADs) alternating with decondensed inter-TADs. Interestingly, these domains display similar distribution pattern across different cell types and developmental stages, i.e. it is a basal feature of the genome [2,6–10]. Insulator proteins have been shown to be central to the establishment and maintenance of this genomic organization, as they may contribute to long-range interaction and counteract the spreading of repressed chromatin state beyond the domain boundary [6,7,10–12].

Numerous efforts have also advanced our understanding of the functional role of morphologically distinct domains seen in the chromosomes under the light microscope: the very well known banding pattern of polytene chromosomes formed by the alternating condensed bands and decondensed interbands has been extensively analyzed and was found to be very similar across different polytene tissues of *Drosophila* (reviewed in [13]).

Recent data allowed combining the information on genome-wide chromatin mapping in *Drosophila* [14,15] with novel approaches to molecularly map the band/interband borders. This has led to the development of the idea that interbands in polytene chromosomes typically correspond to the promoter regions of broadly expressed housekeeping genes, which is in line with the observation that interband regions display “open” chromatin conformation in both polytene chromosomes and in the chromosomes from dividing cells [16–18].

The regions corresponding to inter-TADs were found to be composed of decompacted chromatin and to associate with CHRO protein [7]; this has led to the proposal that interTADs (barriers) and TADs may correspond to interbands and bands, accordingly [19]. Indeed, this proposal has been confirmed experimentally by comparing the molecularly mapped band/interband borders with the positions of TADs [18]. This correlation has been comprehensively analyzed by Eagen et al. [20] who demonstrated that TAD borders matched the flanking regions of large bands found in polytene chromosomes and by Stadler and Eisen [21]. These studies have thus reinforced the hypothesis on the existence of a shared plan of interphase chromosome organization.

This integral view of domain organization of the genome brings together the features of various chromosomal structures described by different methods and opens novel questions to be addressed experimentally. For instance, given that inter-TADs correspond to interbands encompassing housekeeping genes, they should not be simply viewed as barriers. Instead, inter-TADs serve as important functional units that display particular organization related to the ubiquitous expression pattern of the housekeeping genes they host. In this connection we ask what is the interplay between the barrier function and active transcription associated with interband regions.

Insulator proteins are rarely found in the chromosomes without other proteins (in so-called, "alone sites); as a rule they form various associations with other insulator proteins [22,23]. Interbands have been reported to be preferentially associated with BEAF32, dCTCF, CP190, and CHRO as most frequently associated with inter-TADs [6,7,18,24]. BEAF32 and dCTCF have been reported to display DNA-binding activity [25,26], whereas CHRO and CP190 apparently do not associate with DNA directly, and are referred to as “second layer” insulator proteins by Vogelmann et al. [27].

Hence, we asked whether the presence of insulator proteins in the interbands is in any way related to their transcriptional activity or whether it serves as a mere decondensation factor that renders the region permissive to transcription, as is the case of CP190 [28].

To address this question, we took advantage of the UAS–GAL4DBD system and used *Drosophila* polytene chromosomes as a model. First, three typical silent regions 10A1-2, 11A6-9, and 59D1-4 sharing the basic features of IH and appearing in polytene chromosomes as black bands were selected [29, 30]. These regions encompass groups of functionally unrelated genes that remain silent in salivary glands. Further, these regions have been described to be rich in repressive chromatin marks, to lack replication origins and to be associated with specific protein ensembles (constituting BLACK and BLUE chromatin types according to Filion et al., [14] (see also [31–36]). Several flystocks were used that had insertions of multiple UAS elements or UAS-containing transposons in the above three regions. To achieve selective tethering of CHRO and dCTCF to such UAS-harboring bands, transgenic CHRO^GAL4DBD^ or dCTCF^GAL4DBD^ constructs were additionally introduced. Tethering of these proteins into UAS-containing regions has led to the recruitment of other insulator proteins, which resulted in altered local chromatin condensation and protein composition, yet had no detectable effect on transcription or replication timing.

## Materials and methods

### Flystocks and genetics

**Fig 1:**
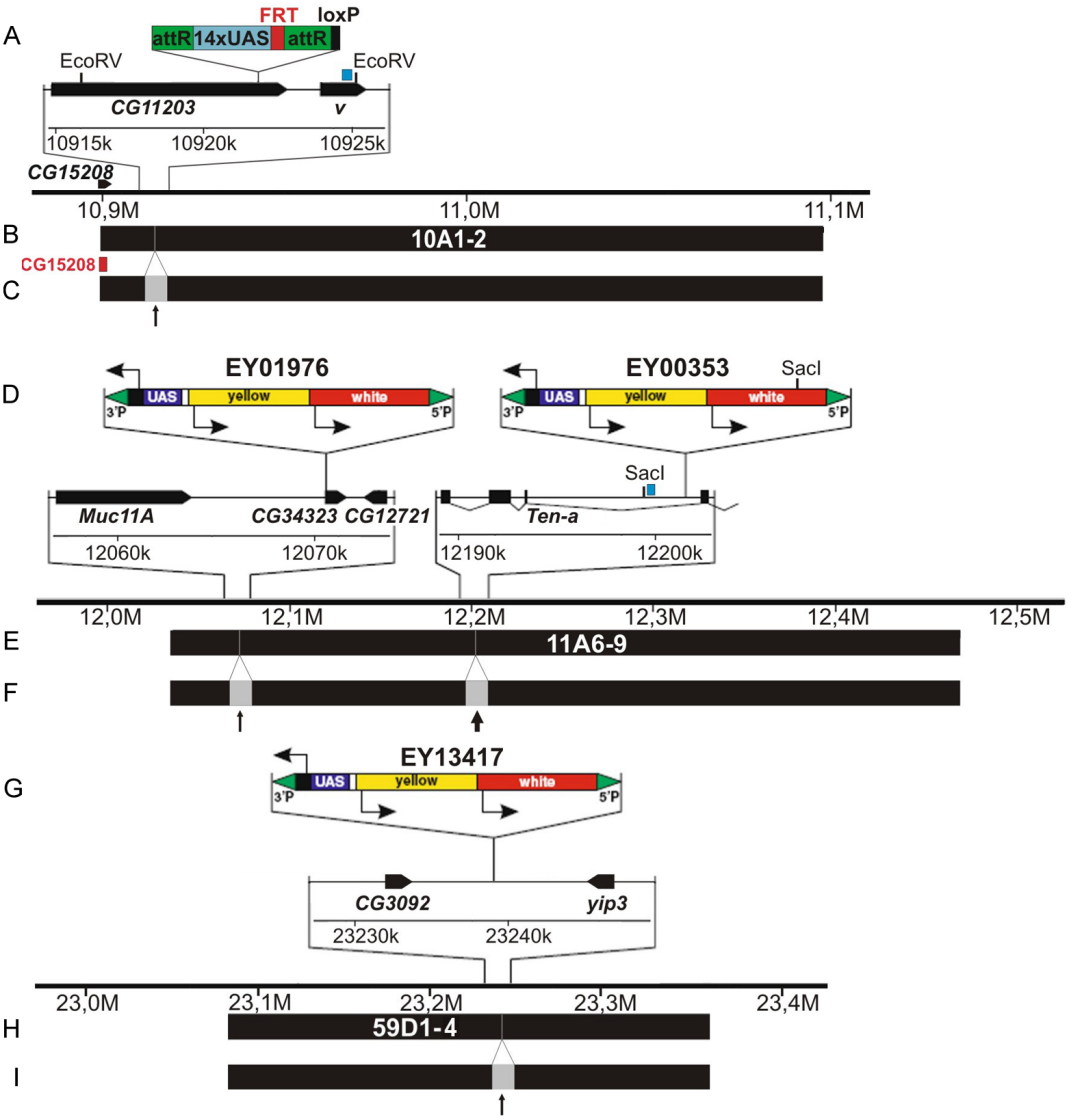
Molecular maps of the genomic regions surrounding the insertions of UAS-bearing transgenes at 10A1-2 (A-C), 11A6-9 (D-F), and 59D1-4 (G-I). A, D, G—organization of 10A-UAS, EY01976, EY00353, and EY13417 transposons and adjacent genes. EcoRV and SacI are the restriction sites used for mapping DHSes in 10A1-2 and 11A6-9 regions, respectively. B, E, H—span of the bands 10A1-2, 11A6-9, 59D1-4 and positions of UAS insertions. C, F, I—schematic illustration of how the bands are expected to split in two fragments separated by an interband (thick and thin arrows) as a result of local CHRO or dCTCF tethering. Red block denotes a fragment of CG15208 gene used for FISH analysis (C).

Previously, we established a fly stock 10A, UAS that contained 14 copies of UAS inserted into the distal part of the band 10A1-2 [37], namely into the 3’UTR of *CG43901* (*CG11203*) (Drosophila genome release 6.06, 10922194) (Fig 1A–1C). Single-copy insertions of EY transposon P(EPgy2) harbor 14xUAS elements and are marked with *yellow*^+^ and *mini-white*^+^ [38]: EY01976 transposon has been mapped in the distal part of the 11A6-9 band within *CG34323* (X: 12070458, r. 6.06); EY00353 has also landed in 11A6-9, but is found in its center, in the intron of *Ten-a* (X: 12203524, r. 6.06) (Fig 1D and 1E); EY13417 maps to the center of 59D1-4 band and is found in the intergenic region between *CG3092* and *yip3* (2R: 23239126, r. 6.06). EY flystocks were kindly provided by the Bloomington Drosophila Stock Center.

We know exactly the position of transpozons, containing array of 14 binding sites for GAL4 in physical map of the bands and know positions of the bands studied [29,36]. Therefore the localization of the chromosome fragment which binds targeted proteins in the central or marginal parts of the bands is easily determined (Fig 1C, 1F and 1I).

We established several stocks allowing heat-shock driven expression of MYC-tagged CHRO^GAL4DBD^ and dCTCF^GAL4DBD^ fusion proteins. As a control, we also used the construct lacking CDSes for CHRO or dCTCF: *hsp70*-GAL4DBD-MYC. Each of these constructs were integrated into the region 51C of the 2R chromosome via phiC31 recombination, which assured identical expression levels of the fusion and control proteins. Cloning details, genetic crosses, as well as functionality tests for ectopic tethering have been described [37]. Distributions of antibodies against fused protein (DBD-Myc-CHRO for DBD-Myc-CTCF) corresponded exclusively to the regions characteristic for both proteins in normal chromosomes (see [37]).

Throughout this study, using standard genetic crosses the flystocks homozygous for both UAS elements and CHRO^GAL4DBD^, dCTCF^GAL4DBD^ or GAL4^DBD^ transgenes were established. In the case of UAS transgenes inserted in 11A6-9, *SuUR* mutant background was additionally introduced, as this allowed complete polytenization and superior morphological visualization of this region [39]. Flies were kept on standard cornmeal-molasses medium at 18–25°C. For polytene chromosome analysis, larvae were grown at 29°C and female third instar larvae were used.

### Fluorescence in situ hybridization (FISH)

Salivary glands were dissected in Ephrussi-Beadle solution, fixed in 3:1 ethanol/acetic acid mixture for about 1 hour at -20°C, and squashed in 45% acetic acid. To remove coverslips, the slides were snap-frozen in liquid nitrogen. Then the slides were stored at -20°C in 70% ethanol. Prior to hybridization, the slides were rehydrated in 2xSSC for 1 hour at 60°C, denatured in 0.07 M NaOH, and passed through a gradient of ethanol series 70-80-96% 5 minutes each and air-dried. Labeled DNA probe was then applied to the slides (biotin-16-dUTP labeling via random-primed polymerase reaction with Klenow fragment. Fragment of the *CG15208* gene was used as a probe (X: 10900830…10901459 bp, r. 6.06), this gene mapping to the edge of 10A1-2 band [17]. Primer sequences to amplify the DNA were as follows: 5’-GCTCTATTGCCGCTGGCTCC-3’; 5’-ACAGATGTCCGGGTGGGGT-3’.

Chromosome squashes were analyzed using epifluorescence optics (Olympus BX50 microscope) and photographed with CCD Olympus DP50.

### Immunostaining of polytene chromosomes

Salivary glands were dissected from third-instar larvae reared at 29°C. Standard procedure for chromosome squashing was followed. Immunostaining was done as described previously [40], with minor modifications. Dilutions of the primary andibodies/antisera were as follows: rabbit polyclonal anti-SUUR [41] (E45; 1:50), mouse monoclonal anti-PCNA (Abcam #ab29; 1:500), rabbit polyclonal anti-CHRO (A.A. Gorchakov) (1:600), mouse anti-Myc (Invitrogen # 46–0307; 1:150), anti-RNA PolII (specific for the Ser5-phosphorylated of Pol II CTD, Covance; 1:50), mouse anti-D1 (gift from Prof. K. McKim, 1:200), rabbit anti-H3K9ac (Abcam, 1:200), goat anti-H3K4me2 (Abcam, 1:100), rabbit anti-WDS (Novus Biologicals, 1:150), mouse anti-H3S10 (Covance, 1:100), antibodies kindly gifted by P.G. Georgiev: rabbit anti-CP190 (1:150), rabbit anti-BEAF-32 (1:100), rabbit anti-dCTCF (1:150), rabbit anti-ZIPIC (1:150), rabbit anti-PITA (1:150), mouse anti-histone H1 and Z4 (provided by Prof H. Saumweber, 1:100 and 1:15, respectively), mouse monoclonal anti-HP1 (CIA9, 1:80) was a gift of Prof. S. Elgin.

Chromosome squashes were incubated with secondary FITC- or rhodamine-labeled goat anti-rabbit and anti-mouse IgG-specific conjugates (Abcam, 1:200), with anti-mouse IgM- FITC conjugates (Sigma, 1:250) or AF488 donkey anti-goat conjugates (Molecular Probes, 1:600). Chromosomes were examined using epifluorescence optics (Olympus BX50 microscope) and photographed with CCD Olympus DP50. Only most typical cases of cytological localizations are shown on the figures in the manuscript in the "Results" section. However, the conclusions are drawn on the basis of analysis of big number of chromosome regions studied (50–100 nuclei with well spread chromosomes in each experiment in several slides). This kind of analysis show reproducibility of localization pattern after IF.

### Mapping of DNase I hypersensitive sites

DNA extraction was done as described elsewhere [42]. Nuclei were isolated as described previously [43]. Equal volumes of nuclei (5 x 10^7^ nuclei) resuspended in buffer A (50 mM Tris–HCl (pH 7.9), 100 mM NaCl, 3 mM MgCl2, 1 mM DTT, and 0.2 mM PMSF) were incubated for 3 min at 37°C with 0, 3, 6 and 12 U of DNase I (Sigma). Reactions were stopped by the addition of EDTA to 25 mM on ice. Following centrifugation and removal of supernatant, the pellets were resuspended in 500 μl of lysis buffer (1.25% SDS, 0.3M Tris–HCl (pH 8.4), 0.1 M EDTA, 5% sucrose), and processed according to the standard phenol/chloroform DNA extraction protocol.

The degree of DNaseI treatment, the completeness of the hydrolysis with the appropriate restriction enzymes, and the amount of DNA in the samples for each line were assessed visually after electrophoresis and gel staining with ethidium bromide.For Southern-blot hybridization experiments equal quantities of DNA samples from each lines treated with 0 and 12 U DNaseI and digested to completion with appropriate restriction enzymes were size-fractionated by electrophoresis on 1% agarose gels in TAE buffer system and transferred on Hybond-XL membrane (Amersham) according to manufacturer’s recommendations.

DNA fragments for 10A1-2 and 11A6-9 regions were obtained by PCR amplification with specific primers for 10A region: 5’- gagttctgacagagcagcgg -3’, 5’- ctgttgggatccaatcatgcg -3’ and 11A region: 5’- gagtagctactcgagcatcg-3’, 5’- gtacaccgctcggtcagac-3’, and used for hybridizations as radiolabeled probes.

## Results

### Ectopic chromosomal tethering of CHRO and dCTCF results in local chromatin decompaction of polytene chromosome bands

Two genetic constructs allowing tethering of the proteins of interest, CHRO^GAL4DBD^ and dCTCF^GAL4DBD^, as well as the control construct encoding Gal4DBD-MYC were used in our experiments. Coding sequences of CHRO and dCTCF proteins were fused in-frame with that of a DNA-binding domain of yeast protein GAL4 (GAL4DBD), tagged with a MYC-epitope for indirect immunodetection purposes and placed downstream of a heat-shock inducible promoter *hsp70*. Given that heat shock is known to affect the chromosomal localization of insulator proteins [44], two induction regimens were tested, namely daily heat shock at 35°C for 30 minutes and entire development at 29°C. Specifically, viability of animals and distribution of chromosomal proteins were assayed in CHRO^GAL4DBD^ and GAL4DBD stocks lacking UAS elements. Both expression conditions resulted in significant pupal lethality of CHRO^GAL4DBD^, but not GAL4DBD animals; larval stages were less sensitive to CHRO^GAL4DBD^ dosage. Hence, we resorted to the strategy of keeping the flies from embryonic up to the pupal stage at 29°C, which allowed virtually normal development and was not accompanied with significant redistribution of chromosomal proteins [45].

To analyze how ectopic tethering of CHRO and dCTCF affects the chromatin structure, three dark bands (10A1-2, 11A6-9, and 59D1-4) harboring insertions of UAS elements were selected. In terms of molecular organization and cytology, these large bands can be classified as typical intercalary heterochromain (IH). This kind of bands is described by the set of characters: dense condensation, late replication and underreplication, ectopic stretches, enrichment with proteins such as H1 histone, SUUR, LAM, D1, and enrichment with genes of development repressed in the salivary glands ([29–36] and many others).

#### 10A1-2 band

**Fig 2:**
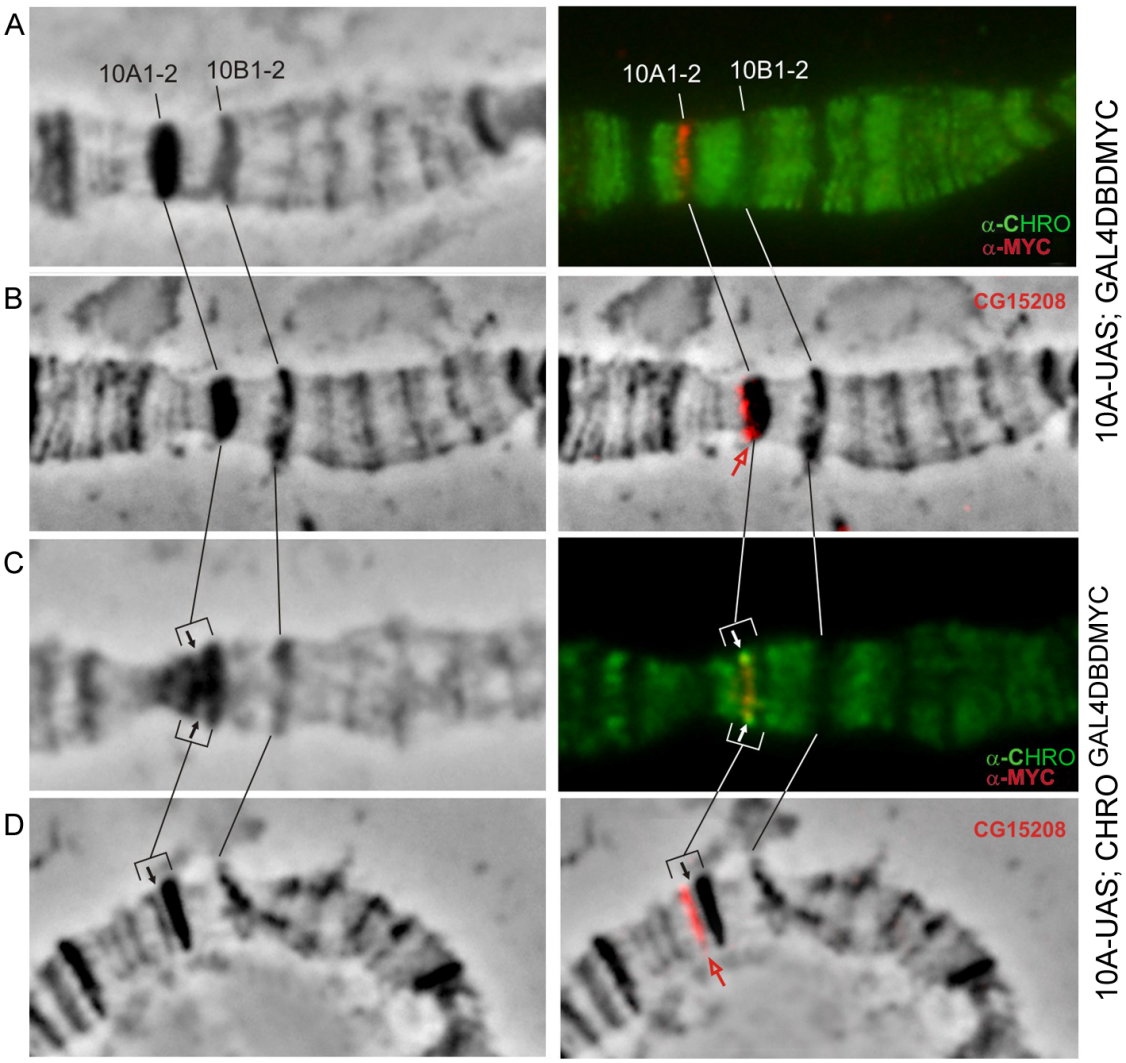
10A1-2 band splits upon CHRO^GAL4DBD^ tethering. Phase contrast (left column). Overlay of phase contrast and immunostaining (A, C) or FISH (B, D) signals (right column). Black and white arrows point to the tethering region, red arrow indicates the position of *CG15208* on the edge of band in control chromosomes (B) or in the distal fragment that has split from 10A1-2 upon tethering (D). CHRO (green), MYC (red), FISH signal (red).

At both 25°C and 29°C, CHRO is found exclusively in the interbands of polytene chromosomes (Fig 2A, see as well S1 and S6 Figs). (On this and the next Figures results only for the bands 10A1-2 and 11A6-9 are shown. In the supplementary material, in addition to the merged images we provide all channels of the immunostaining and FISH experiments).

In the control chromosomes from 10A-UAS-GAL4DBD-MYC female larvae, MYC-signal marking the tethering site (shown as red in Fig 2A) localizes to the distal part of 10A1-2 and shows no overlap with the signal for endogenous CHRO. FISH using *CG15208* probe which maps upstream of the UAS-element (Fig 1A, Vatolina et al., [17]) is consistent with localization in the distal part of 10A1-2 (red in Fig 2B). Upon CHRO tethering to this region, distal part of 10A1-2 forms a thin grey band that has split away from the band thereby giving rise to a prominent decondensed zone on the edge of 10A1-2 (marked as arrows in Fig 2C and 2D). CHRO- and MYC-specific signals display co-localization within this decondensed region (yellow in Fig 2C). In these conditions, FISH with *CG15208* probe indicates that the split grey band harbors the sequence of CG15208 (Fig 2B–2D and S1 Fig). Thus, the band 10A1-2 was found to split, and a novel thin band is indicated by a black arrow in the Fig 1C and 1D.

#### 11A6-9 band

**Fig 3:**
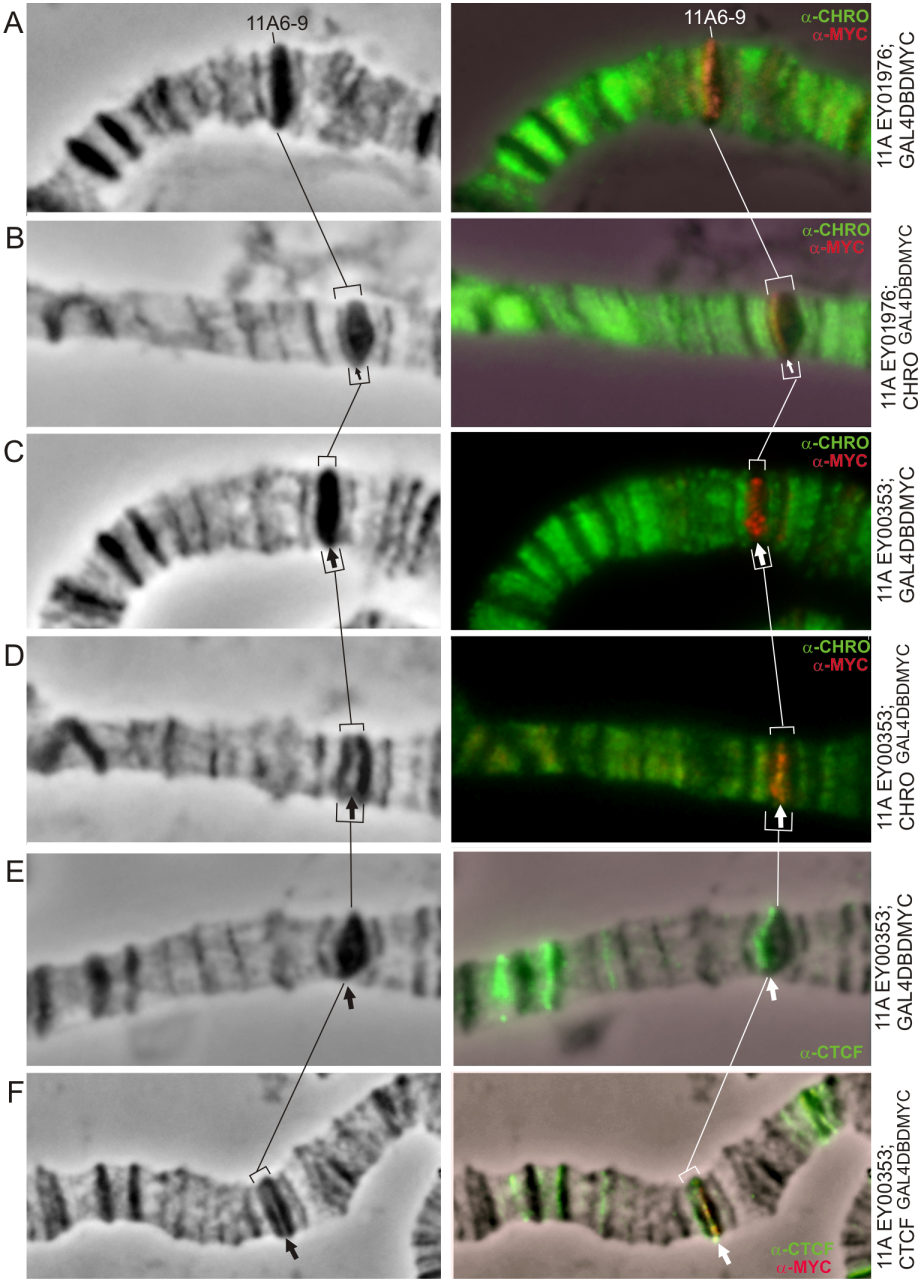
11A6-9 band splits upon tethering of CHRO^GAL4DBD^ (A-D) and dCTCF^GAL4DBD^ (E,F) into the regions of EY01976 (A,B) and EY00353 (C-F) insertions. Phase contrast (left column). Overlay of phase contrast and immunostaining (right column). Thin arrow indicates EY01976 insertion (A—control, B—CHRO^GAL4DBD^ expression and splitting of the band 11A6-9 in its distal part), thick arrow indicates EY00353 insertion in the middle of the band (C,E—control; D,F—tethering of CHRO^GAL4DBD^ and dCTCF^GAL4DBD^, respectively).

In wild-type animals, the band 11A6-9 is known to be the region that replicates extremely late [31], and in females the vast majority of DNA sequences close to the center of the band remain heavily underpolytenized, which manifests cytologically as a polytene chromosome break or constriction. In contrast, in *SuUR* mutants this region is fully polytenized [39] and appears as one of the most prominent bands on the X chromosome (Fig 3A–3C and 3E and S3 and S4 Figs), therefore to facilitate visualization the band the *SuUR* mutant background was introduced into stocks studied. This mutation results in faster replication fork progression, with the chromatin composition and the sequence of replication origin firing remaining unchanged [34].

In the case of the distally inserted UAS-transposon EY01976 (Fig 1D and 1E), ectopic tethering of CHRO (Fig 3A and 3B) resulted in splitting of a thin grey band from the distal part of 11A6-9 concomitant with formation of a decondensed region separating this grey band and the remainder of 11A6-9. CHRO and MYC signals co-localized in this decondensed region (thin arrow in Fig 3B). In the control chromosomes (GAL4DBD recruitment only), no structural changes were apparent, nor CHRO was detectable in this region (Fig 3A). Similarly, in the absence of CHRO tethering (i.e. upon GAL4DBD recruitment), insertion of the EY00353 element in the center of 11A6-9 band (Fig 1D and 1E) does not alter the band morphology, nor the distribution of endogenous CHRO (Fig 3C). In contrast, upon CHRO^GAL4DBD^ tethering, the 11A6-9 band breaks into two parts separated by a decompacted interband-like zone. This is accompanied with an overlap between CHRO and MYC signals (Fig 3D).

In polytene chromosomes from EY00353 (GAL4DBD-MYC) larvae, localization of endogenous dCTCF is restricted to just a few sites in the region 10A -11A6-9 (Fig 3E), with one of these sites being distal to 11A6-9. dCTCF is normally absent from both 10A1-2 and 11A6-9 bands. Upon ectopic expression of dCTCF^GAL4DBD^, the 11A6-9 band splits in two fragments with an intervening decompacted region (thick arrow in Fig 3F) becoming positive for MYC and dCTCF. Morphological changes observed in different tethering conditions in the band 11A6-9 are schematically illustrated in Fig 1E and 1F.

#### 59D1-4 band

**Fig 4:**
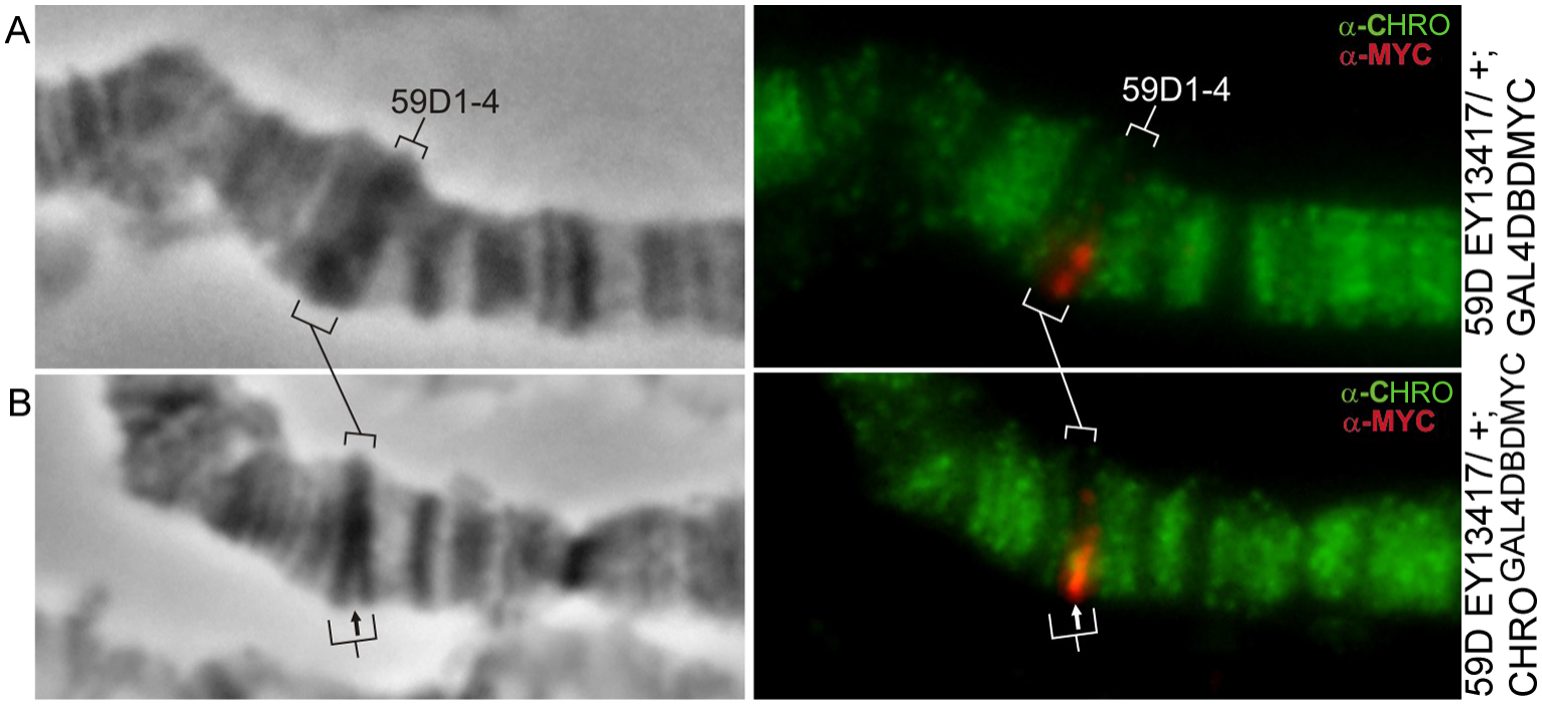
59D1-4 band splits in the heterozygotes for EY13417 insertion upon CHRO^GAL4DBD^ tethering. Control EY13417/+; GAL4DBD chromosomes (A). Tethering of CHRO^GAL4DBD^ to one UAS-bearing homolog manifests as a partial splitting of the 59D1-4 band in its central part (B).

The EY13417 insertion lies in the middle of the 59D1-4 band (Fig 1G and 1H), and upon ectopic CHRO^GAL4DBD^ tethering to this UAS-containing element, we observed very similar scenario to those described above. Due to the localization of both EY13417 and CHRO^GAL4DBD^-encoding transgenes on the second chromosome, the chromosomes from animals trans-heterozygous for these insertions were analyzed, and the band 59D1-4 was found to split on just a single homolog (Figs 4B, 1H and 1I and S3 Fig). In the site of splitting both signals upon CHRO and MYC are seen.

To summarize, using three distinct intercalary heterochromatic bands as a model, we showed that ectopic recruitment of CHRO and dCTCF results in the formation of a decompacted, interband-like, structure.

### Active chromatin marks become detectable in the sites of ectopic CHRO tethering

**Fig 5:**
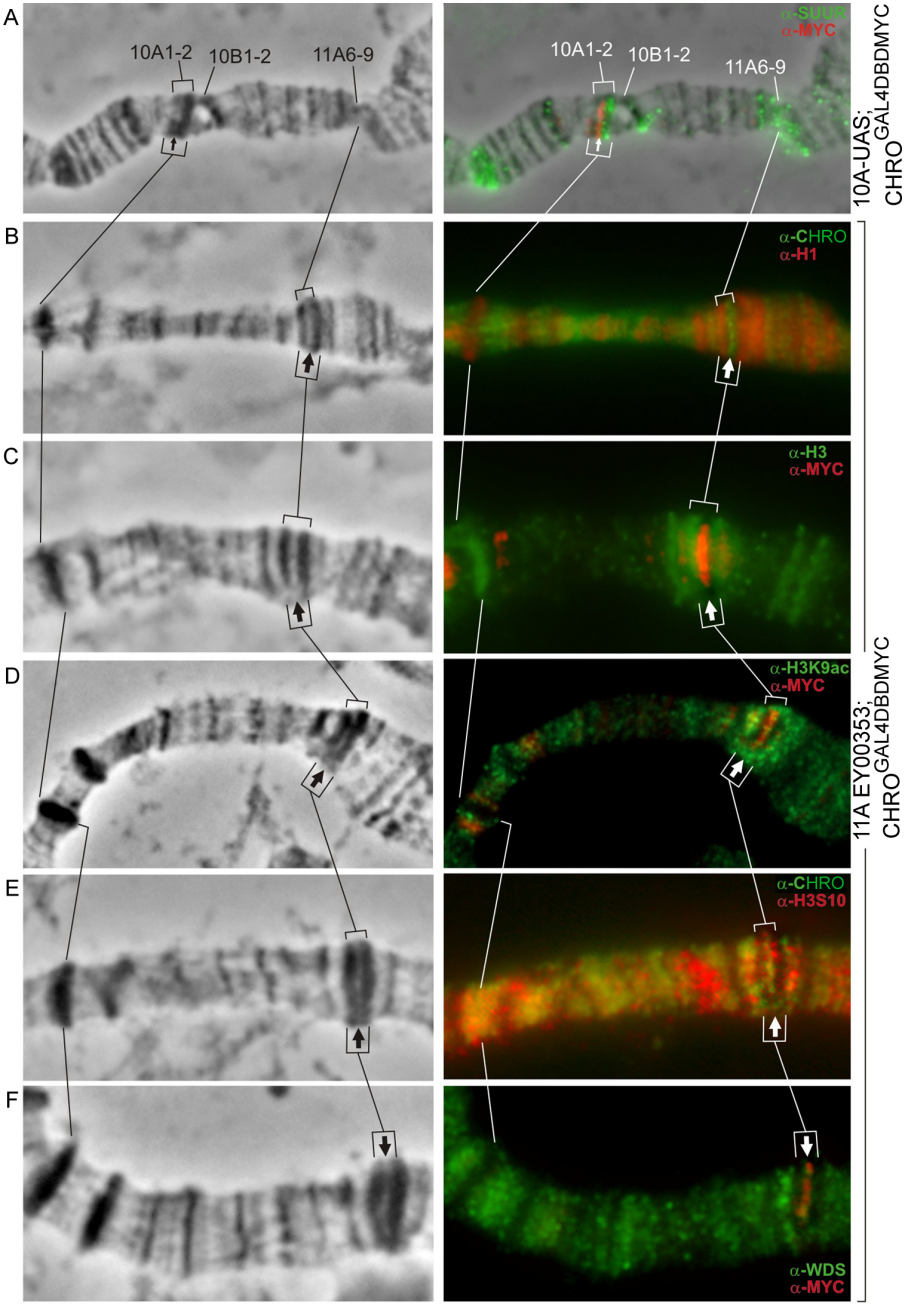
Epigenetic make-up of the newly formed interbands in the context of 10A1-2 and 11A6-9 bands. Phase contrast (left column); overlay of the phase contrast and immunostaining signals for SUUR (A), H1 (B), H3(C), H3K9ac (D), H3S10 (E), WDS (F) (right column). Thin and thick arrows denote the novel interbands formed at UAS sites in the bands 10A1-2 and 11A6-9, accordingly.

First, we asked whether chromatin decompaction induced by ectopic CHRO tethering would be associated with the loss of repressive chromatin marks. We focused our analysis on just two bands, 10A1-2 and 11A6-9, and the data for the UAS insertion in the middle of 11A6-9 are shown (Fig 5 and S4 Fig, Table 1). Previously, histone H1, SUUR, and histone H3 were demonstrated to be present in the dark intercalary heterochromatic bands [46], which is consistent with the observations made in our study. Each of the bands– 10A1 or 11A6-9—lacking UAS elements served as an internal control for the UAS-harboring band. In the absence of ectopic CHRO recruitment (GAL4DBD expression only), histones H1, H3, as well as heterochromatic markers D1 and SUUR displayed uniform staining of 10A1-2 and 11A6-9 band material. Upon CHRO^GAL4DBD^ tethering, H1 and H3 signals as well as D1 (not shown) localized exclusively to the compact regions of 11A6-9 that flanked the recruitment site, i.e. these signals were not detectable in the decompacted zone (arrows in Fig 5B and 5C). SUUR localization was analyzed only in the context of the region 10A1-2, in the chromosomes of 10A,UAS- CHRO^GAL4DBD^ animals, as they carried *SuUR+* background. SUUR was present only in the compact portion of the split band, and decompaction zone lacked detectable SUUR binding (Fig 5A).

**Table 1:** Summary of protein immunodetection data in the chromosomes from CHRO^GAL4DBD^- and dCTCF^GAL4DBD^-expressing animals.

Proteins	Presence (+) or absence (-) of binding in the targeting sites	
	CHRO^GAL4DBD^	dCTCF^GAL4DBD^
**Silent chromatin proteins**: histones H1 and H3; D1, SUUR	–	–
**Open chromatin marks and proteins**: H3K9ac, H3K4me2, H3S10, WDS	+	+
**Insulator proteins**:		
CHRO	+	+
CTCF	–	+
BEAF-32, PITA, ZIPIC,	–	–
Z4	+	+
CP190	+	+

Next, we asked whether loss of repressive marks would be accompanied with the acquisition of proteins or histone modifications typical of the open chromatin. These include RNA pol II, H3K9ac, H3S10, and histone acetyltransferase WDS [16,18]. These proteins were absent from the bands 10A1-2 and 11A6-9 upon expression of the control GAL4DBD protein. Upon CHRO^GAL4DBD^ recruitment in either 10A1-2 or 11A6-9 context, H3K9ac, H3S10, and WDS became recruited in addition to CHRO (Fig 5D, 5E and 5F).

**Fig 6:**
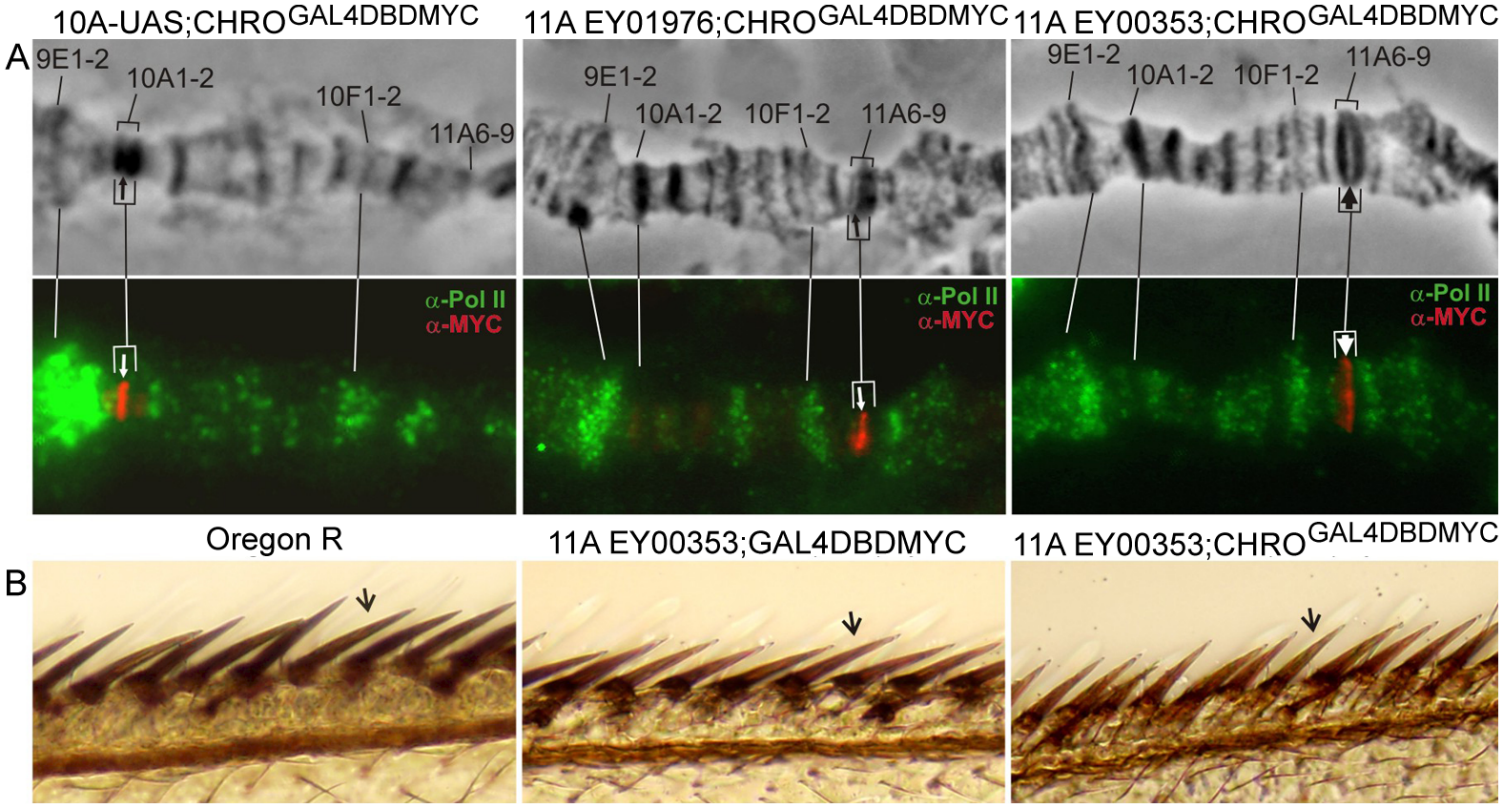
Ectopic tethering of CHRO^GAL4DBD^ does not lead to transcription of the decompacted chromatin. (A)–anti-MYC signal (UAS) and anti-RNA PolII Ser5 signal do not co-localize in the decompacted interband-like regions formed in the bands 10A1-2 and 11A6-9. Upper row: phase contrast of the split-band morphology, bottom row: immunostaining signals for RNA PolII Ser5 (green) and MYC (red). Positions of UASes are denoted by arrows; (B)–wing bristle pigmentation in *Oregon* R (dark), EY00353; DBDGAL4 (brown) and EY00353; CHRO^GAL4DBD^ (brown) flies indicates that the reporter *yellow*^+^ gene present in EY elements is not induced upon CHRO^GAL4DBD^ tethering.

Given that RNA polII is a typical interband protein [18] and that CHRO tethering into IH bands led to the opening of chromatin in the UAS sites, we asked whether this was also accompanied with the transcription of these regions. To address this question, immunostaining of chromosomes with antibodies against phosphoSer5 RNA pol II was performed. Fig 6A demonstrates that the presence of novel open chromatin regions in 10A1-2 and 11A6-9 regions was not associated with detectable RNA pol II signals. Only MYC signal marking the binding of the CHRO^GAL4DBD^ fusion protein was present. At the same time, many of the normally decompacted regions of polytene chromosomes, such as ecdysone-induced puffs 9E1-2 and 10EF found near these bands were RNA pol II-positive (Fig 6A).

EY00353 transgene inserted in the center of 11A6-9 has two reporter genes, *yellow*+ and *mini-white*+ near the UAS element. EY00353 flies display partial silencing of these reporters by the surrounding repressive chromatin, and so this results in their mosaic expression in the eyes and bristles [46]. For instance, some of the wing bristles in these flies appear much lighter than those in wt, and this mutant pigmentation was restored to normal levels upon induction of expression with a strong and ubiquitous tub-Gal4 driver [46]. Hence, we proceeded to the analysis of how CHRO-mediated chromatin decompaction may affect the expression of reporter genes. Pigmentation of bristles on anterior wing margin was compared between three genotypes: *Oregon R* (wild type), EY00353; GAL4DBDMYC, and EY00353; CHRO^GAL4DBDMYC^. Regardless of whether GAL4DBD or CHRO^GAL4DBD^ protein was recruited to UAS elements upstream of *yellow+* reporter, these wing bristles remained lighter than in wt, which we interpret as the failure of expression induction in the targeting region (Fig 6B). The same was observed for the second reporter, *mini-white*+, in eyes and testes (data not shown). We did not find activation of reporter genes expression in UAS-construct upon targeting dCTCF as well.

Overall, CHRO^GAL4DBD^ and dCTCF^GAL4DBD^ tethering led to the replacement of silent chromatin marks with open chromatin marks. However, this chromatin decondensation and opening the chromatin was not accompanied with induction of transcription.

### CHRO and dCTCF recruit other insulator proteins

Recent genome-wide analyses indicate that insulator proteins are frequently found as ensembles that may have distinct functional properties, depending on the exact composition [11,22,23,47,48]. For instance, inter-TADs have been reported to be co-occupied by CP190, BEAF-32, CHRO, and dCTCF [6,7]. Given that recruitment of CHRO and dCTCF led to band splitting and formation of a novel interband-like structure, we asked whether other insulator proteins would also become detectable in this region. So far, several well-known insulator proteins—CHRO, dCTCF, Z4, CP190 (see above), as well as the recently described PITA and ZIPIC insulator proteins targeting CP190 to chromatin [49] have been tested.

**Fig 7:**
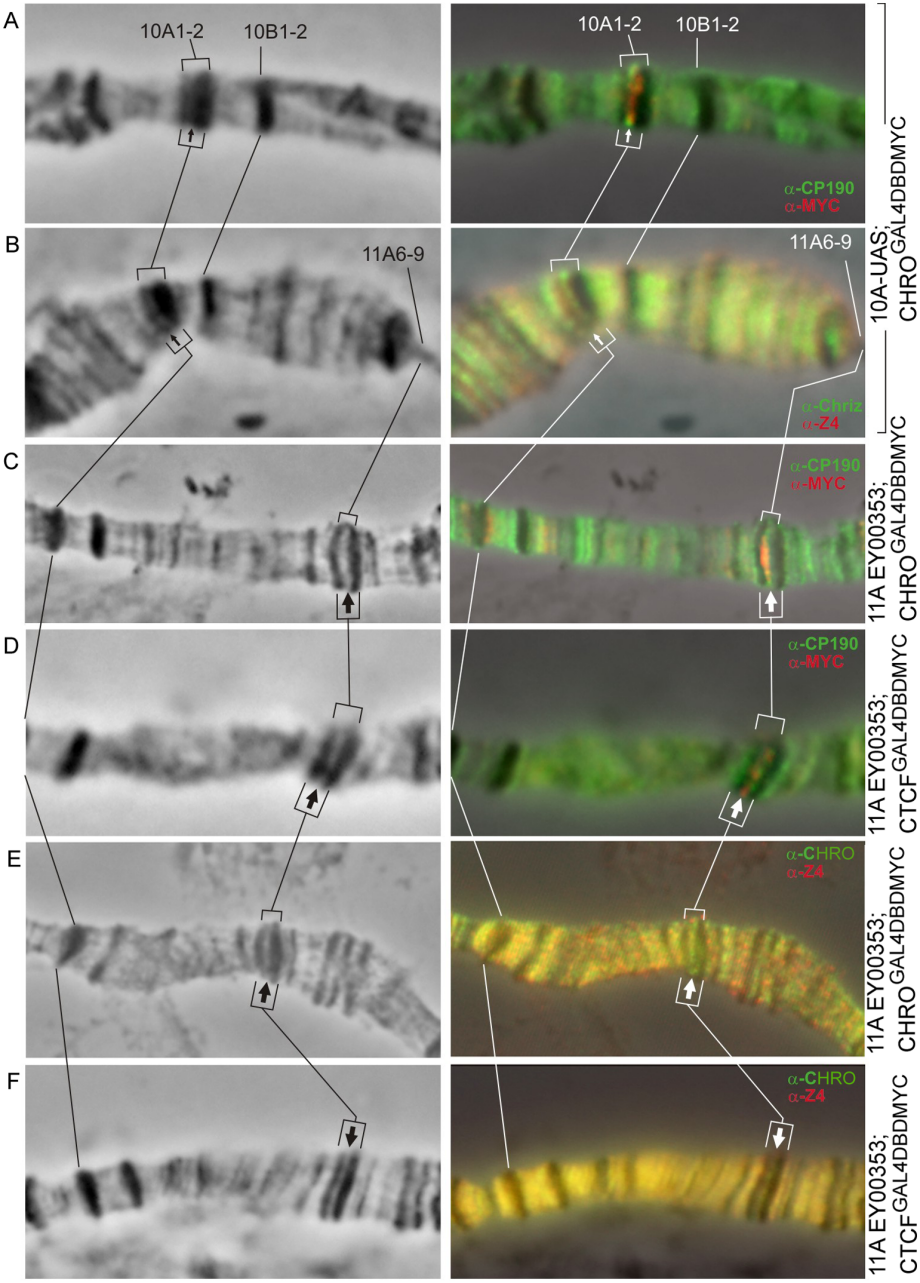
Immunodetection of insulator proteins in the decompacted regions formed within the bands 10A1-2 (A, B) and 11A6-9 (C–F). Left column: 10A1-2 and 11A6-9 region of the X chromosome (phase contrast), right column: overlay of phase contrast and immunostaining data. Arrows indicate the position of decompaction zone within 10A1-2 (thin arrows) and 11A6-9 (thick arrows) bands.

Both CHRO and dCTCF were found to recruit CP190 and Z4. In addition, dCTCF recruits CHRO. At the same time BEAF, PITA, ZIPIC were not detected in the targeting regions (Table 1, Fig 7, S5 and S6 Figs).

### Mapping of DNase I hypersensitive sites (DHSes)

We used indirect end labeling technique to map DHSes in the chromatin of UAS-containing regions in the bands 10A1-2 and 11A6-9. Material prepared from flies with GAL4DBD-MYC or CHRO^GAL4DBDMYC^ background was used.

**Fig 8:**
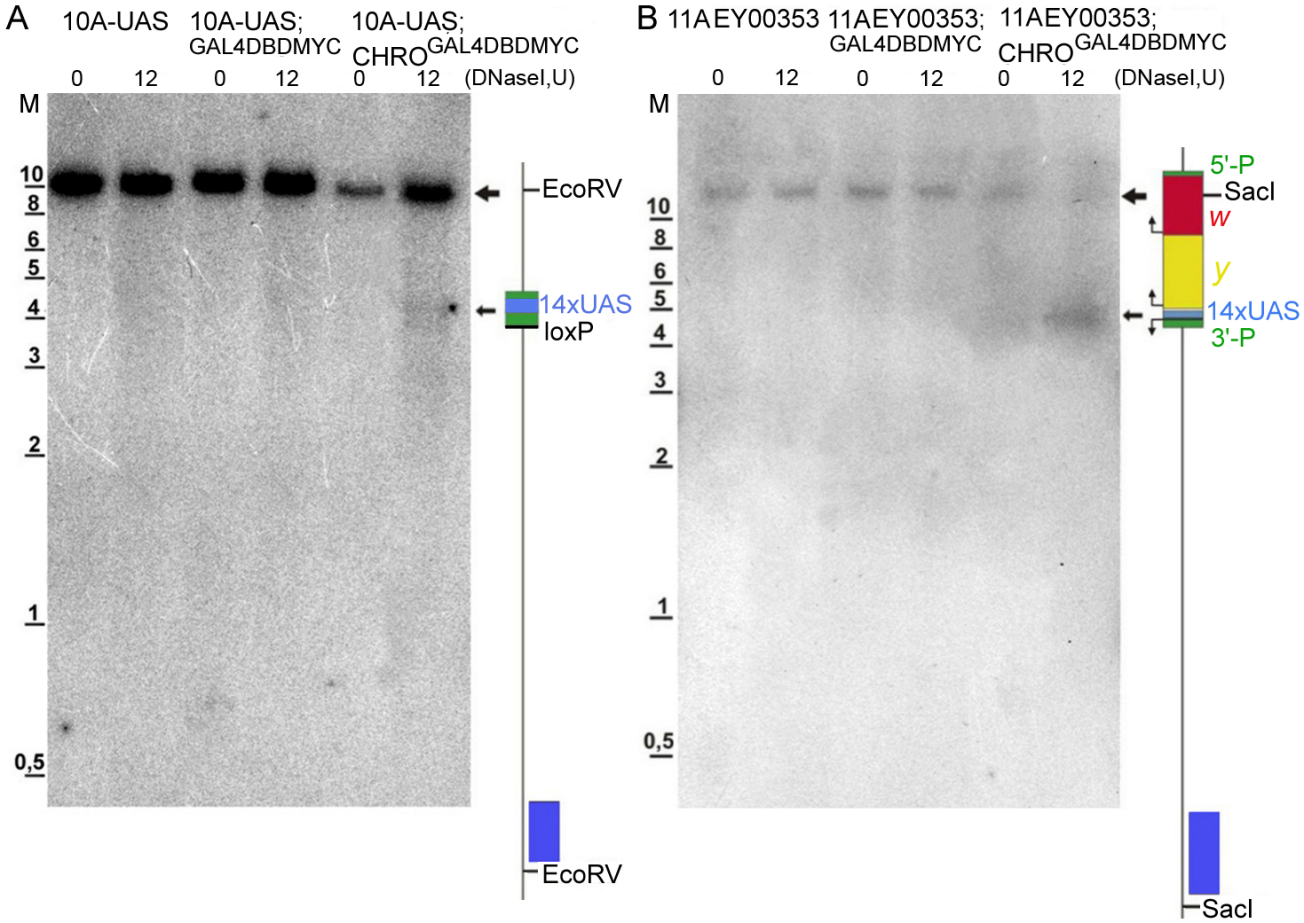
DHS mapping in the UAS region within the bands 10A1-2 and 11A6-9. (A) Southern-blot hybridization data for the probe from the region 10A1-2 with the *Eco*RV-treated DNA isolated from DNaseI-digested (0 U and 12 U) nuclei from third-instar larvae of the following genotypes: 10A,UAS; 10A,UAS GAL4DBD-MYC; and 10A,UAS CHRO^GAL4DBD^. Map of the 10.3 kb EcoRV fragment encompassing the 14xUAS cassette integrated in the region 10A1-2 is shown on the right. (B) Southern-blot hybridization data for the probe from the region 11A6-9 with the *Sac*I-treated DNA isolated from DNaseI-digested (0 U and 12 U) nuclei from third-instar larvae of the following genotypes: EY00353; EY00353 GAL4DBD-MYC; and EY00353 CHRO^GAL4DBD^. Map of the 12,3 kb fragment encompassing part of the EY transposon and adjacent genomic DNA. Large arrows point to the hybridization signals corresponding to the full-length *Eco*RV-fragment from the region 10A1-2 and to the *Sac*I-fragment from the region 11A6-9; small arrows indicate the DHSes detectable in the regions of interest upon expression of chimeric proteins and mapping to the 14xUAS module. Positions of the probes used for indirect-end labeling are shown as blue bars. M—molecular weight marker (kb).

Chromatin from animals having UAS multimers integrated into 10A1-2 was treated with DNase I, and besides the only hybridization signal clearly visible in all analyzed samples (Fig 8A), which well corresponds to expected full-length 10.3 kb EcoRV hybridization fragment (Fig 1A). Smeared hybridization signal was also present in the chromatin isolated from GAL4DBD-MYC-expressing 10A-UAS flies; importantly the smear became less pronounced and specific bands appeared upon CHRO^GAL4DBDMYC^ expression. It is only in this situation (10A-UAS; CHRO^GAL4DBDMYC^) that a prominent DHS was observed to cover a ~400 bp fragment that maps to the region spanning 14xUAS repeats.

When EY00353 animals expressing either control GAL4DBD-MYC or CHRO^GAL4DBDMYC^ proteins were used as a source of chromatin, the hybridization pattern within the 12.3 kb SacI DNA fragment encompassing genomic fragment and most of the EY material (Fig 1D) was essentially similar to the situation described above for the band 10A1-2. Very little to no nuclease activity is detectable in EY00353-only animals. It is slightly increased upon GAL4DBD-MYC expression, whereas in EY00353; CHRO^GAL4DBDMYC^ animals, a prominent DHS of ~400 bp is visible in the region corresponding to the 14xUAS cassette (Fig 8B). Upon DNase I treatment of the purified DNA (instead of the nuclei), no specific signals are observed (data not shown).

Our data, therefore, support the idea that the presence of DHSes in the UAS-regions within 10A1-2 and 11A6-9 bands upon tethering of CHRO^GAL4DBDMYC^ to these sites is associated with altering the chromatin context towards euchromatinization.

### Ectopic tethering of CHRO and dCTCF does not affect the replication timing of decompacted regions formed in the bands

It is well-established that decondensed regions such as interbands are the first to begin replication in the S phase. Densely packed IH bands, in contrast, are known to complete replication the last.

Hence, we were curious to know whether the replication dynamics would be altered in any way in the condensed band upon formation of an “open” chromatin island within it. This was studied for the regions 10A1-2 and 11A6-9, with anti-PCNA immunostaining as a tool to monitor ongoing replication [45,46].

**Fig 9:**
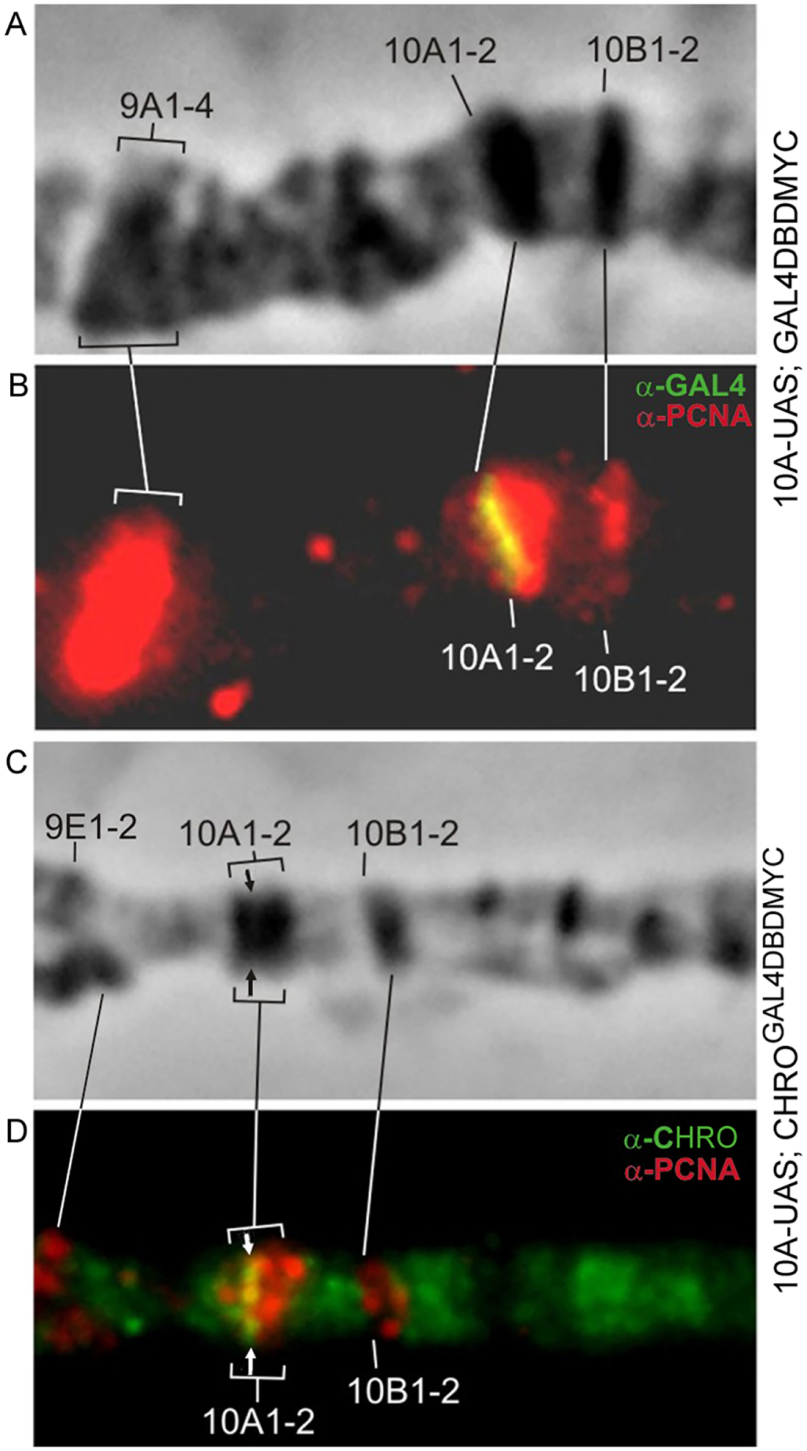
Replication timing in the band 10A1-2. Control 10A-UAS; GAL4DBD chromosomes (A,B), 10A-UAS; CHRO^GAL4DBD^ (C,D), CHRO (green), PCNA (red). Arrow indicates the decondensation site.

**Fig 10:**
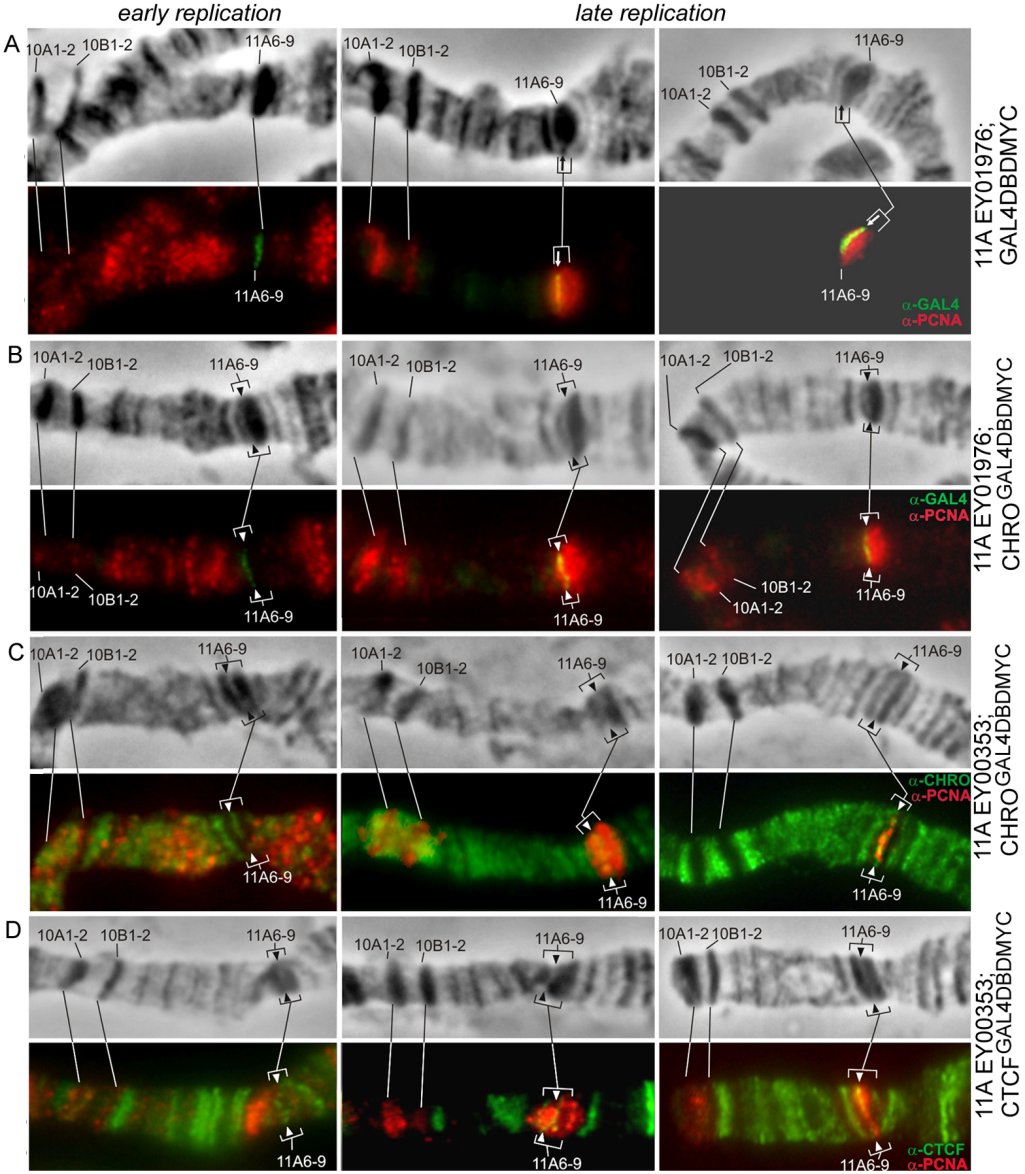
Replication timing in the band 11A6-9. Control—EY01976; GAL4DBD chromosomes (A), YE01976; CHRO^GAL4DBD^ (B), GAL4 (green), PCNA (red), EY00353; CHRO^GAL4DBD^ (C), CHRO (green), PCNA (red), EY00353, dCTCF^GAL4DBD^, dCTCF (green), PCNA (red) (D). Arrows point to the position of UAS repeats and to the decompaction zone.

In 10A-UAS; CHRO^GAL4DBD^ chromosomes, the decompacted region embedded in the 10A1-2 is separated from the adjacent interband by a novel thin band (Fig 1C). Late replication in the chromosome region 9A-10B is shown in Fig 9B. Only a handful of bands—9A1-4, 10A1-2, and 10B1-2, known as extremely late-replicating regions of the genome [31] appear positive for PCNA. In control animals (10A-UAS; GAL4DBD-MYC) UAS sequences replicate simultaneously with the rest of the 10A1-2 sequences, i.e. very late in the S phase (Fig 9A and 9B). Using same-stage chromosomes from 10A-UAS, CHRO^GAL4DBD^ animals, we saw that decompacted material remained late-replicating (arrow in Fig 9D). Replication dynamics can be monitored in greater detail in the region 11A6-9: it is so large that early and late replication stages can be conveniently distinguished. At early stages, PCNA signal is detectable across the chromosome, except for the late-replicating bands, such as 10A1-2, 10B1-2, 11A6-9 [46]. Much as in the wild-type situation, in the chromosomes from EY01976; GAL4DBD animals, replication schedule for the UAS-region remains unaffected (Fig 10A, green signal on the left hand images). Later in the replication, only 10A1-2, 10B1-2, and 11A6-9 bands remain PCNA-positive (including the very UAS cassette) (Fig 10A, yellow signal in the central column). In the very end of replication, only the chromocenter and just several bands (one of which is 11A6-9) are associated with PCNA (Fig 10A, yellow signal in the right column). Replication dynamics remains unchanged upon CHRO^GAL4DBD^ tethering, as the decompacted region enters replication late, similarly to the wild-type situation (Fig 10B, column on the left). At later stages, it actively replicates along with the rest of the 11A6-9 band (Fig 10B, yellow signal in the right column).

When CHRO^GAL4DBD^ is targeted to EY00353 inserted in the middle part of the band 11A6-9, decondensed material of the transgene remains PCNA-negative in early S phase (Fig 10C, left column). Later, when 10A, 10В, and 11A6-9 bands replicate, it replicates as well (Fig 10C, central column). In late S phase, when 10A and 10В have finished replication, this material still shows PCNA signal (Fig 10C, right column). The same scenario is observed upon dCTCF^GAL4DBD^ tethering (Fig 10D), namely, targeting site remains as late-replicating as in the control.

Taken together, these results demonstrate that local chromatin decondensation induced by tethering of CHRO or dCTCF in the late-replicating bands of polytene chromosomes does not result in sooner completion of replication at these sites.

## Discussion

In polytene chromosomes, interbands appear as decondensed regions; these sites bring together two important functions as they host the promoters of housekeeping genes and replication origins. Further, interbands are known to separate TADs (densely packed bands of polytene chromosomes), which in all likelihood necessitates that interbands also carry a barrier function to prevent the silencing chromatin state of bands from spreading. This functional complexity of interbands is matched with their rich and complex epigenetic profile. These genomic regions are the hot spots for transcription factors, for the assembly of replication machinery and, as was described recently, for insulator proteins.

Permanent decondensation of interbands may be related to the constant transcription of the housekeeping genes they host. Nonetheless, it is unlikely that transcription *per se* is sufficient to provide this level of decondensation. Apparently, there should be other decondensation factors involved, such as insulator proteins. In this regard, the study by Ahanger et al. [28] is of particular interest, as tethering of CP190 insulator protein was shown to decondense a compact lacO array in both mammalian and fruitfly chromosomes. “Chromatin-opening” function of insulators is further supported by the positive correlation observed between the presence of insulator proteins and nucleosome depletion [23,28,50 and references therein].

In the present study, we asked how insulator proteins may affect the chromatin structure. To answer this question, we used an artificial GAL4/UAS-based recruitment system to tether CHRO and dCTCF to a single genomic site embedded in an intercalary heterochromatin environment and to analyze the accompanying chromatin changes.

CHRO (also known as CHRIZ) was originally identified as an interband-specific protein [24,51]. It was later described as a *bona fide* insulator protein, as it contributed to the establishment or maintenance of long-range interactions in complex with a prominent insulator protein BEAF [27]. CHRO and BEAF are highly enriched in the interbands [17,18]; DNA-binding dCTCF protein is not invariably present in the interbands, yet it functions as a hub for interactions with other insulator proteins.

Our analyses show that CHRO and dCTCF tethering to UAS elements placed in bands 59D1-4, 10A1-2, and 11A6-9 is accompanied with local chromatin decondensation and formation of interband-like structures. This effect was observed regardless of the position—central or side-shifted—of the UAS element within these bands. Epigenetic make-up of the region also changed significantly, as repressive chromatin marks (H1, H3, D1, and SUUR) characteristic of the compact bands were gone, whereas open chromatin markers—H3K4me2, H3K9ac, H3S10, and WDS, characteristic of interbands—would appear. It must be kept in mind that in the wild-type situation and upon tethering of the control GAL4DBD protein, open chromatin marks are never detectable in these regions. Tethering the fused protein GAL4DBDHP1 to the insertion EY00353 does not result in splitting the 11A6-9 (S3E Fig). Therefore the tethering any large protein (functional or not) will not lead to a disruption of structure if it is not a protein of open chromatin. Altered chromatin composition caused by tethering of insulator proteins is also supported by the observed increase in nuclease sensitivity of these regions.

Of special interest is that chromatin decondensation in our system is not accompanied with recruitment of RNA polymerase II. Lack of local transcription in the artificial interbands thus formed is consistent with the unchanged status of expression of the two marker genes, *yellow*^+^ and *mini*-*white*^+^, situated immediately adjacent to the UAS elements. In the original flystock carrying EY00353 insertion in 11A6-9, the flies display mosaic eye color as well as wing bristle pigmentation [46]. This phenotype is caused by the mosaic inactivation of marker genes by the surrounding silent chromatin of the band. Upon GAL4-mediated activation of EY00353, the phenotype becomes more wild-type (red eyes, darker bristles) (Fig 3 in Koryakov et al., [46]). In contrast, recruitment of chimeric CHRO and dCTCF proteins, even under strong heat-shock induction conditions, had no effect on the degree of pigmentation of wing bristles (Fig 6B). Chromatin appears to become poised for expression but fails to recruit the components of transcriptional machinery. Our results are in line with the observations that CP190-mediated chromatin decondensation was not associated with local transcription activation [28]. The situation when chromatin is poised for transcription, yet does not transcribe, appears to be quite frequent. For instance, chromatin of IH bands has been described to harbor tiny regions of open chromatin lacking nonetheless detectable transcription of the underlying genes. These regions were notably rich in insulator proteins and behaved as hubs concentrating such proteins [36]. Multiple other studies have also reported chromatin decondensation in the absence of transcription [52–55].

Importantly, neither our data nor the data published by other groups show that the effects of insulators observed here are direct: tethering the proteins of interest is invariably accompanied with recruitment of other insulator proteins. For instance, CHRO tethering brings CP190 and Z4 (the primary chromosomal partner of CHRO), but not BEAF or dCTCF to the ectopic site; dCTCF, in turn, attracts CHRO, CP190, and Z4.

Taken together, these data are consistent with the idea that besides many other functions played by the insulator proteins, they also have a role in keeping the chromatin open, as this is a prerequisite for the permanent activity of the interband-resident housekeeping genes. In other words, insulators may be viewed as the elements that block the silent chromatin of bands from spreading into interbands. Yet, insulator proteins are not the only players contributing to the induction of open chromatin state, as they are known to be assisted by other factors having chromatin-decondensing activities. Upon tethering of CHRO and dCTCF, the H3S10 histone mark appears in the region, which suggests the recruitment of JIL-1 kinase, the enzyme having interband-specific distribution pattern and directly associating with CHRO [56]. CHRO and JIL-1 are together required for the maintenance of normal polytene chromosome structure: JIL-1 tethering results in histone H3S10 phosphorylation, decompaction of densely packed chromatin regions acquiring interband-like morphology, which is not associated with their transcription [57–59]. In our experiments, tethering of CHRO and dCTCF leads to the recruitment of WDS, a component of chromatin remodeling machinery. This indicates that chromatin-opening functions of insulators are associated with a complex network of other factors, and the exact details of these interactions remain to be described.

Our data indicate that in the absence of transcription, chromatin decondensation is insufficient for altering the replication timing. The current consensus in the field is that in the *Drosophila* genome, replication is initiated in the decondensed regions of interbands having high density of replication origins. Replication forks then bidirectinally enter the body of adjacent bands where they ultimately converge. The rate of replication fork progression may depend on how tightly the chromatin of bands is packed, and is known to be affected by the SUUR protein. Clearly, this scenario implies that edges of the bands enter replication soon after the interbands, and so within each band a center-wise replication gradient thus forms. In other words, replication time along the chromatin fiber is a function of the distance from the band’s edge [36]. This model is based on the genome-wide ORC2 mapping data and the correlations with the dynamics of replication timing across different parts of the bands [29,32,34,36,60]. Thus, the edges of the bands typically complete replication before the body of most bands does. The region that replicates the last in the band is typically found in its center, which is manifested by the strongest degree of underreplication in this zone. From this standpoint, one unit of replication referred to as a replicon is composed of a “band+interband” pair, and so each replicon features a full gradient of replication timing ranginf from very early to very late. This pattern changes substantially, once a decondensed and actively transcribed region is formed within the band (such as via GAL4-mediated activation of UAS constructs), and this region becomes early-replicating [46]. A similar situation is also observed naturally, when the genes embedded into the silent chromatin of the dense bands become developmentally activated: this manifests as small islands of active RED chromatin appearing in transcriptionally inert BLACK and BLUE chromatin types (5-color chromatin model [14]). Notably, this is accompanied by the acquisition of an early replication pattern by these RED regions [29]. Other examples of coordinated replication and transcription are also known (reviewed by [61,62].

In our study, additional evidence was obtained to support the interplay between replication and transcription. Upon CHRO or dCTCF tethering to the center of 11A6-9, a decondensed region lacking detectable transcription forms. In this system, decondensed chromatin state was insufficient for shifting local replication timing, as it remained late, much as without tethering. Taken together, these results support the functional coordination between replication and transcription programs [63–66].

## Supporting information

S1 Fig 10A1-2 band splits upon CHRO^GAL4DBD^ tethering. Immunostaining (A,B) and FISH (C, D) signals. Each column (A-D) shows—phase contrast (PH) for the fragment of the X chromosome (subdivision 10), immunostaining and overlay of immunostaining (from upper to bottom row, consequently). Left columns (A,C) show tethering GAL4DBD-MYC (control), right columns (B,D) show splitting the 10A1-2 band upon CHRO^GAL4DBD^ tethering. Black and white arrows point to the decondensed region, red arrow indicates the position of *CG15208* on the edge of band in control chromosomes (C) or in the distal fragment that has split from 10A1-2 upon tethering CHRO (D). CHRO is shown in green, MYC—red, FISH signal is red. Lines connect homologous regions of the chromosomes. Bar represents 5 μm.
(TIF)

S2 Fig 11A6-9 band splits upon tethering of CHRO^GAL4DBD^ (A-D) into the regions of EY01976 (A,B) and EY00353 (C,D) insertions. Each column (A-D) shows: phase contrast (PH), immunostaining and overlay of immunostaining (from upper to bottom row, consequently). Lines connect homologous regions of the chromosomes. Upper columns indicate EY01976 insertion (A—control, B—CHRO^GAL4DBD^ expression and splitting of the band 11A6-9 in its distal part), bottom columns indicate EY00353 insertion in the middle of the band (C—control; D—tethering of CHRO^GAL4DBD^ and splitting of the band 11A6-9 in its central part). The arrows point to the decompacted regions.
(TIF)

S3 Fig 59D1-4 band splits in the heterozygotes for EY13417 insertion upon CHRO^GAL4DBD^ tethering (A,B) and splitting the 11A6-9 band in strain with EY00353 (C-E). Control EY13417/+; GAL4DBD-MYC chromosomes (A); tethering of CHRO^GAL4DBD^ to one UAS-bearing homolog manifests as a partial splitting of the 59D1-4 band in its central part (B). The 11A6-9 band splits upon tethering dCTCF^GAL4DBD^ in EY00353 insertion in its central part (C,D). Control EY00353;GAL4DBDMYC, normal pattern binding of dCTCF protein in 10A1-2—11A region (C). Tethering of dCTCF^GAL4DBD^ and splitting band 11A6-9, the new binding site with dCTCF protein marks its decompacted part (D). Tethering HP1^GAL4DBD^ [67] in EY00353 insertion does not split 11A6-9 band (E). Each column (A-E) from upper to bottom row, consequently, shows—phase contrast (PH), immunostaining and overlay of immunostaining, consequently. Lines connect homologous regions of the chromosomes and the arrows point to the decondensed regions.
(TIF)

S4 Fig Epigenetic make-up of the newly formed interbands in the context of the 10A1-2 and 11A6-9 bands. Immunostaining signals for SUUR (A), H1 (B), H3 (C), H3K9ac (D), H3S10 (E), WDS (F). The arrows denote the novel interbands formed at UAS sites in the bands 10A1-2 and 11A6-9, respectively. Chromatin marks dense chromatin go away (A-C), and active marks appear instead (D-F). Each column (A-E) shows—phase contrast (PH), immunostaining and overlay of immunostaining from upper to bottom row, consequently.
(TIF)

S5 Fig Immunodetection of insulator proteins in the decompacted regions present around and formed within the bands 10A1-2 (A,B) and 11A6-9 (C-E). Insulator proteins CP190, CHRO and Z4 become associated with tethering of CHRO^GAL4DBD^ in the regions of UAS-10A (A,B) and EY00353 (C-D) insertions, but dCTCF protein was undetectable upon CHRO^GAL4DBD^ tethering in decompacted region (E). Each column (A-E) shows—phase contrast, immunostaining and overlay of immunostaining (from upper to bottom row, consequently). Arrows indicate the position of decompacted zone within 10A1-2 and 11A6-9 bands.
(TIF)

S6 Fig Immunodetection of insulator proteins with tethering of CHRO^GAL4DBD^ and dCTCF^GAL4DBD^ in EY00353 insertions within 11A6-9 band. BEAF32, ZIPIC and PITA proteins were undetectable in decompacted regions after tethering of CHRO^GAL4DBD^ (A-C). Tethering of dCTCF^GAL4DBD^ induced binding of CP190, CHRO and Z4 proteins (D,E), but not BEAF32 protein (F). Each column (A-E) shows—phase contrast, immunostaining and overlay of immunostaining (from top to bottom, consequently). Arrows indicate the position of decompacted zone within 11A6-9 band.
(TIF)

S1 Text Supplementary text. (DOC)
