# Supplementary figures and images for: Tethering of CHROMATOR and dCTCF proteins results in decompaction of condensed bands in the Drosophila melanogaster polytene chromosomes but does not affect their transcription and replication timing
Source: PLoS One. 2018 Apr 2;13(4):e0192634. doi: 10.1371/journal.pone.0192634 (PMC5880345; doi:10.1371/journal.pone.0192634)

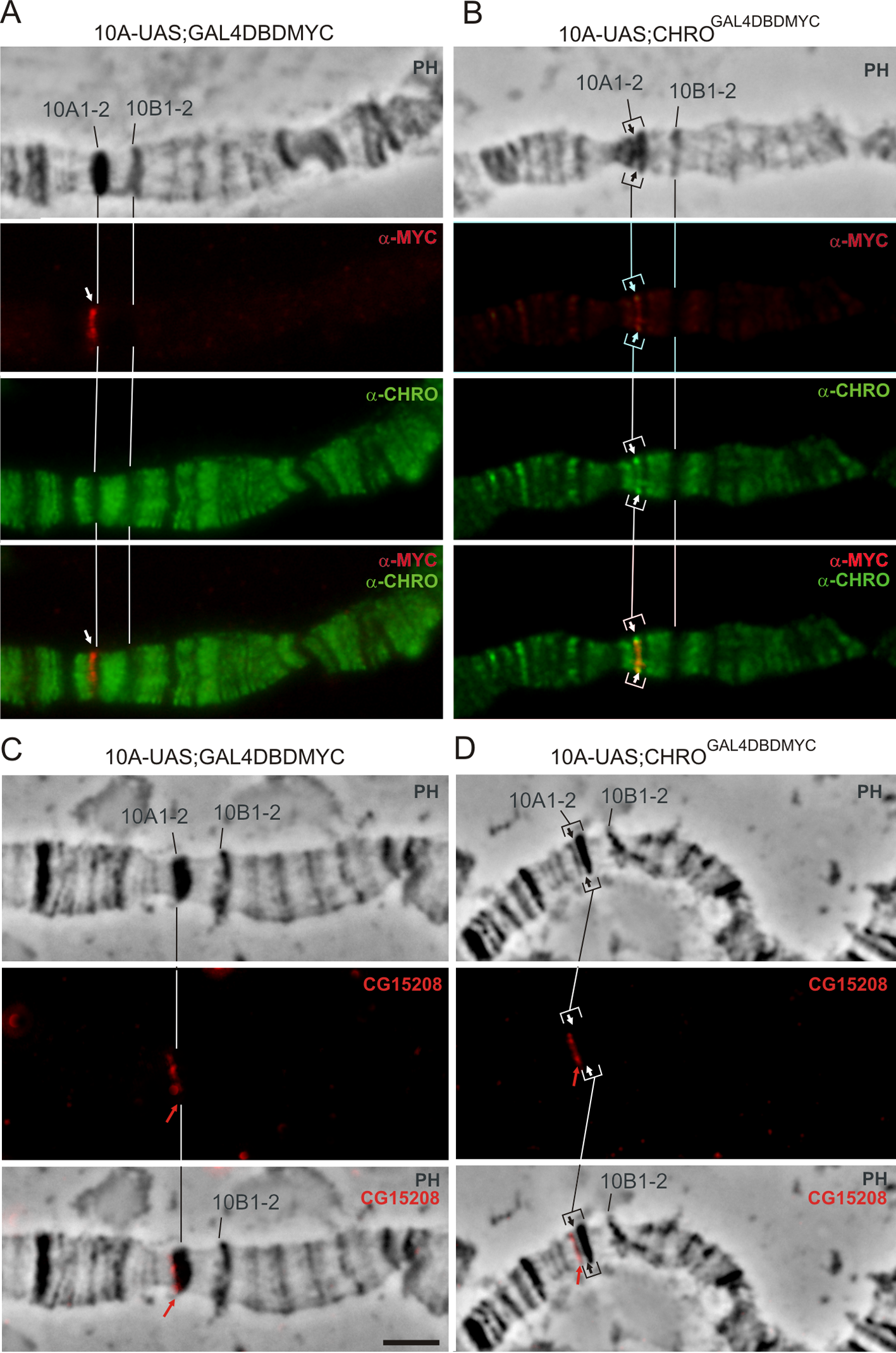

Supplement: S1 Fig — Immunostaining (A,B) and FISH (C, D) signals. Each column (A-D) shows—phase contrast (PH) for the fragment of the X chromosome (subdivision 10), immunostaining and overlay of immunostaining (from upper to bottom row, consequently). Left columns (A,C) show tethering GAL4DBD-MYC (control), right columns (B,D) show splitting the 10A1-2 band upon CHROGAL4DBD tethering. Black and white arrows point to the decondensed region, red arrow indicates the position of CG15208 on the edge of band in control chromosomes (C) or in the distal fragment that has split from 10A1-2 upon tethering CHRO (D). CHRO is shown in green, MYC—red, FISH signal is red. Lines connect homologous regions of the chromosomes. Bar represents 5 μm. (TIF) [file pone.0192634.s001.tif]

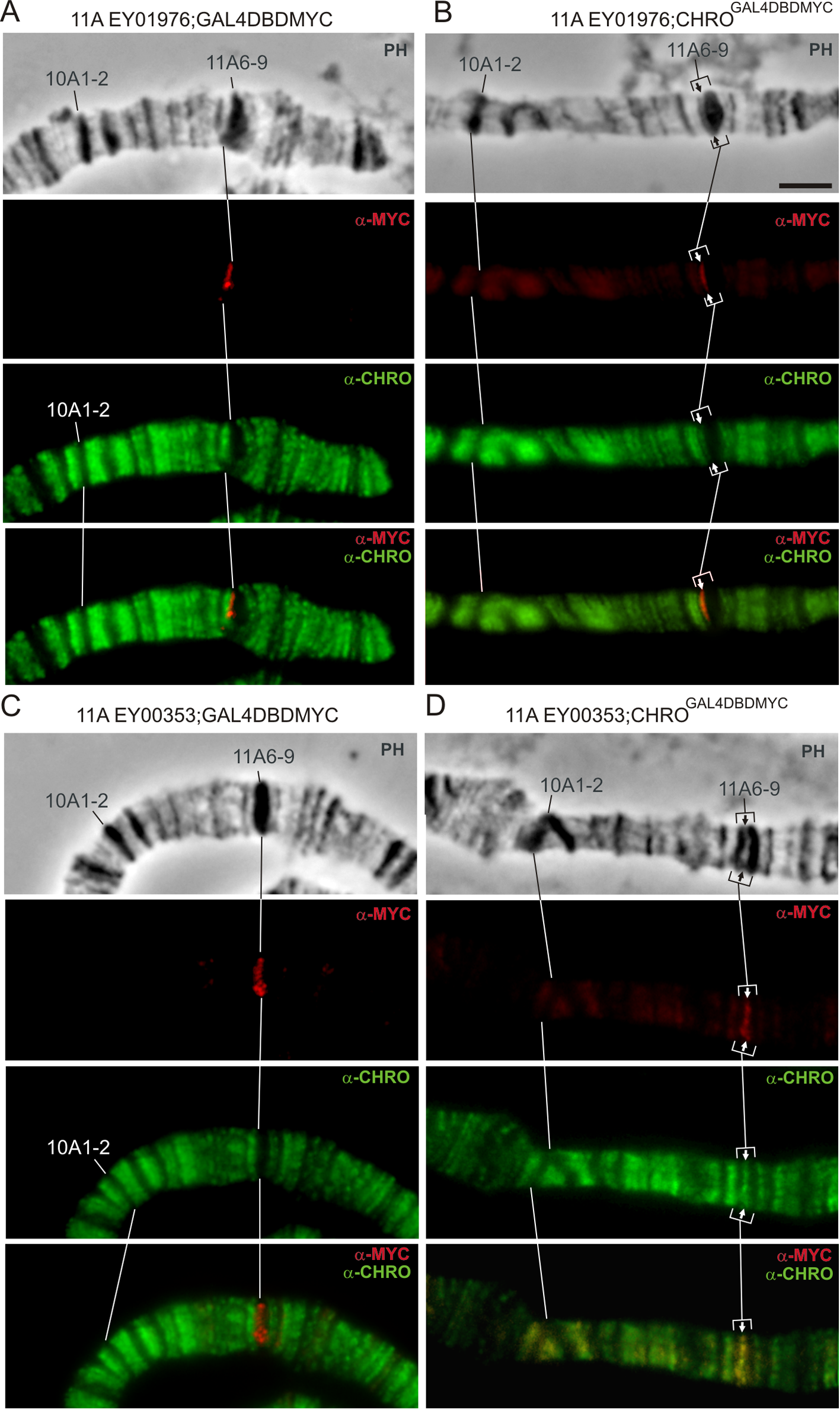

Supplement: S2 Fig — Each column (A-D) shows: phase contrast (PH), immunostaining and overlay of immunostaining (from upper to bottom row, consequently). Lines connect homologous regions of the chromosomes. Upper columns indicate EY01976 insertion (A—control, B—CHROGAL4DBD expression and splitting of the band 11A6-9 in its distal part), bottom columns indicate EY00353 insertion in the middle of the band (C—control; D—tethering of CHROGAL4DBD and splitting of the band 11A6-9 in its central part). The arrows point to the decompacted regions. (TIF) [file pone.0192634.s002.tif]

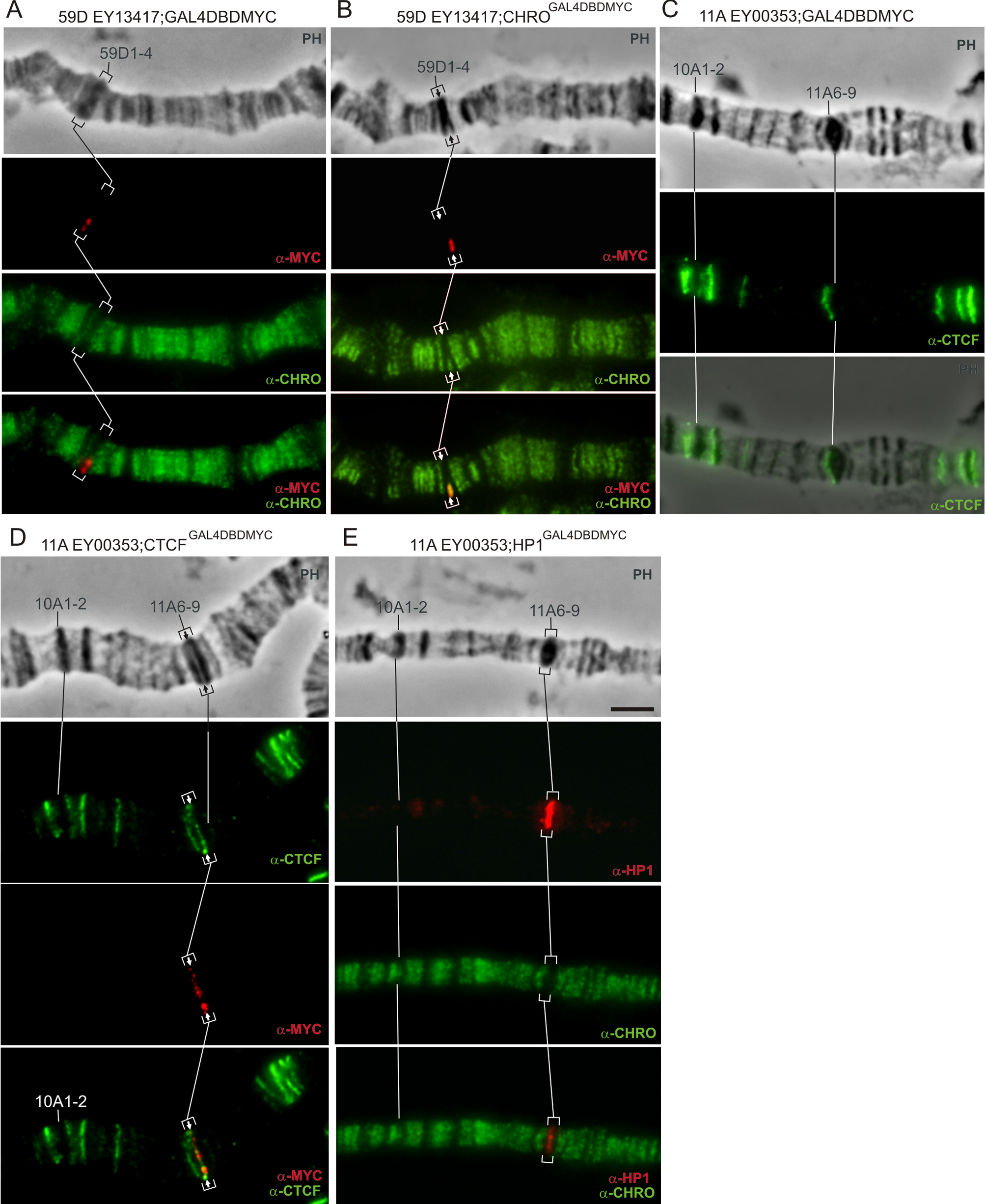

Supplement: S3 Fig — Control EY13417/+; GAL4DBD-MYC chromosomes (A); tethering of CHROGAL4DBD to one UAS-bearing homolog manifests as a partial splitting of the 59D1-4 band in its central part (B). The 11A6-9 band splits upon tethering dCTCFGAL4DBD in EY00353 insertion in its central part (C,D). Control EY00353;GAL4DBDMYC, normal pattern binding of dCTCF protein in 10A1-2—11A region (C). Tethering of dCTCFGAL4DBD and splitting band 11A6-9, the new binding site with dCTCF protein marks its decompacted part (D). Tethering HP1GAL4DBD [67] in EY00353 insertion does not split 11A6-9 band (E). Each column (A-E) from upper to bottom row, consequently, shows—phase contrast (PH), immunostaining and overlay of immunostaining, consequently. Lines connect homologous regions of the chromosomes and the arrows point to the decondensed regions. (TIF) [file pone.0192634.s003.tif]

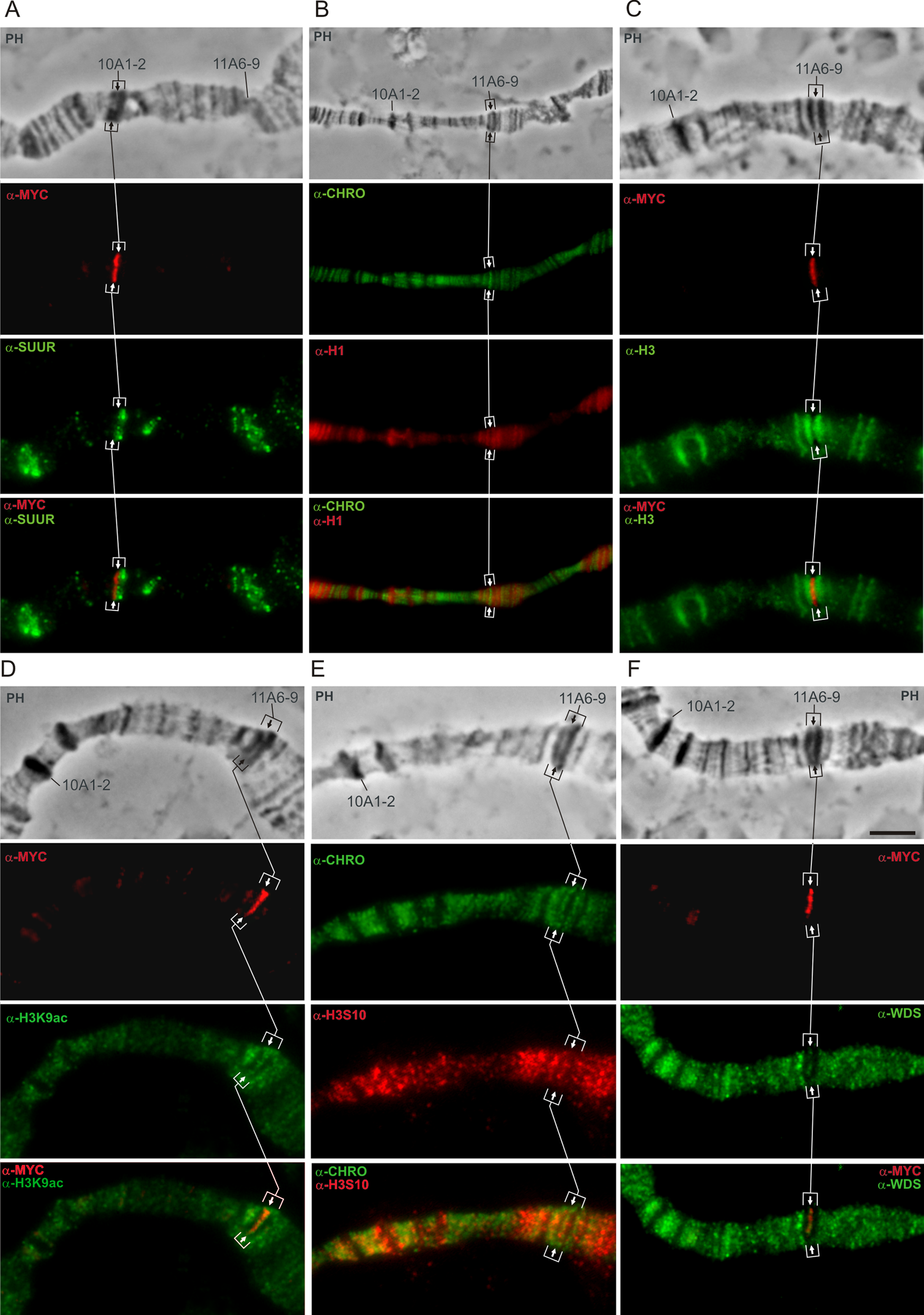

Supplement: S4 Fig — The arrows denote the novel interbands formed at UAS sites in the bands 10A1-2 and 11A6-9, respectively. Chromatin marks dense chromatin go away (A-C), and active marks appear instead (D-F). Each column (A-E) shows—phase contrast (PH), immunostaining and overlay of immunostaining from upper to bottom row, consequently. (TIF) [file pone.0192634.s004.tif]

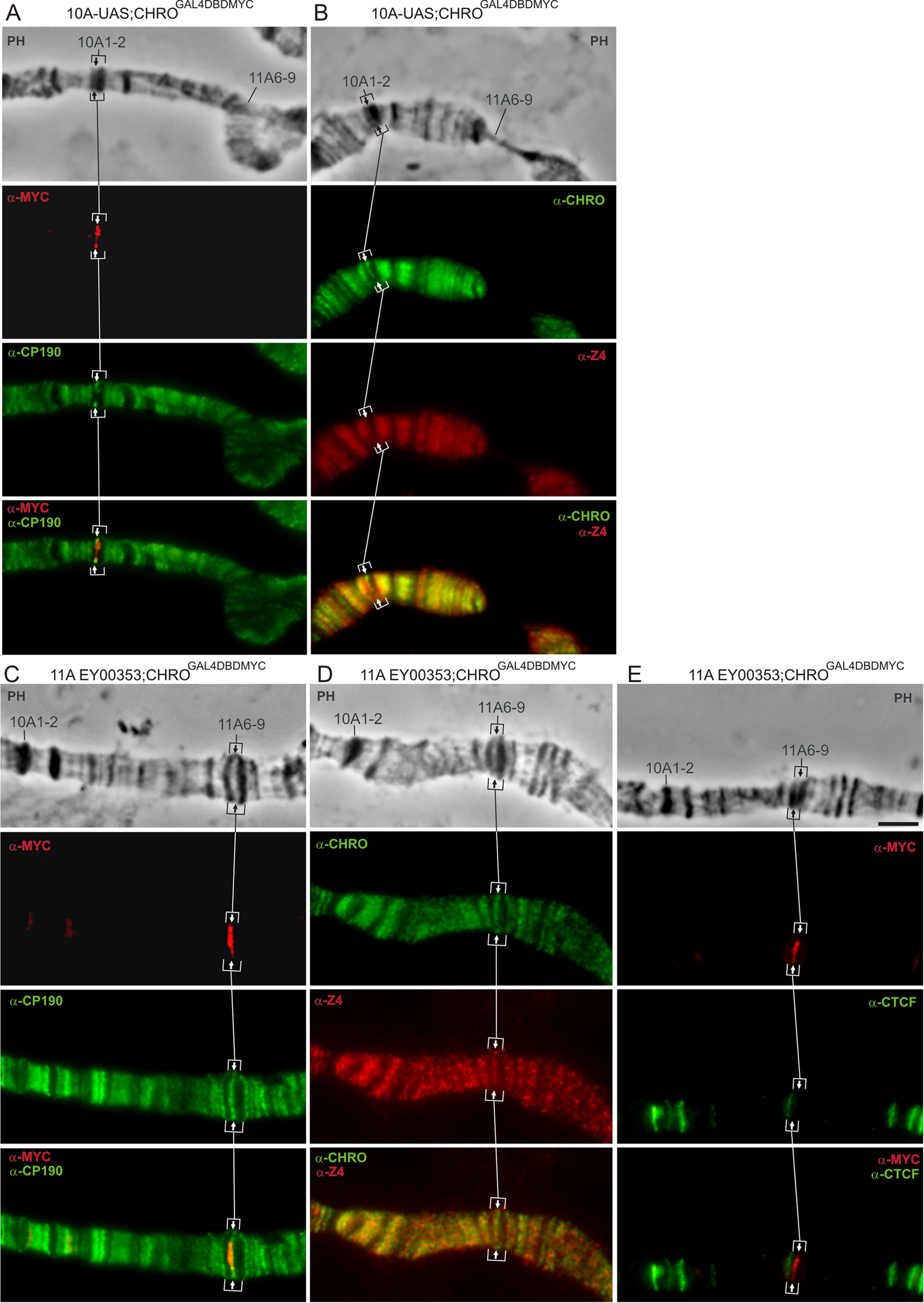

Supplement: S5 Fig — Insulator proteins CP190, CHRO and Z4 become associated with tethering of CHROGAL4DBD in the regions of UAS-10A (A,B) and EY00353 (C-D) insertions, but dCTCF protein was undetectable upon CHROGAL4DBD tethering in decompacted region (E). Each column (A-E) shows—phase contrast, immunostaining and overlay of immunostaining (from upper to bottom row, consequently). Arrows indicate the position of decompacted zone within 10A1-2 and 11A6-9 bands. (TIF) [file pone.0192634.s005.tif]

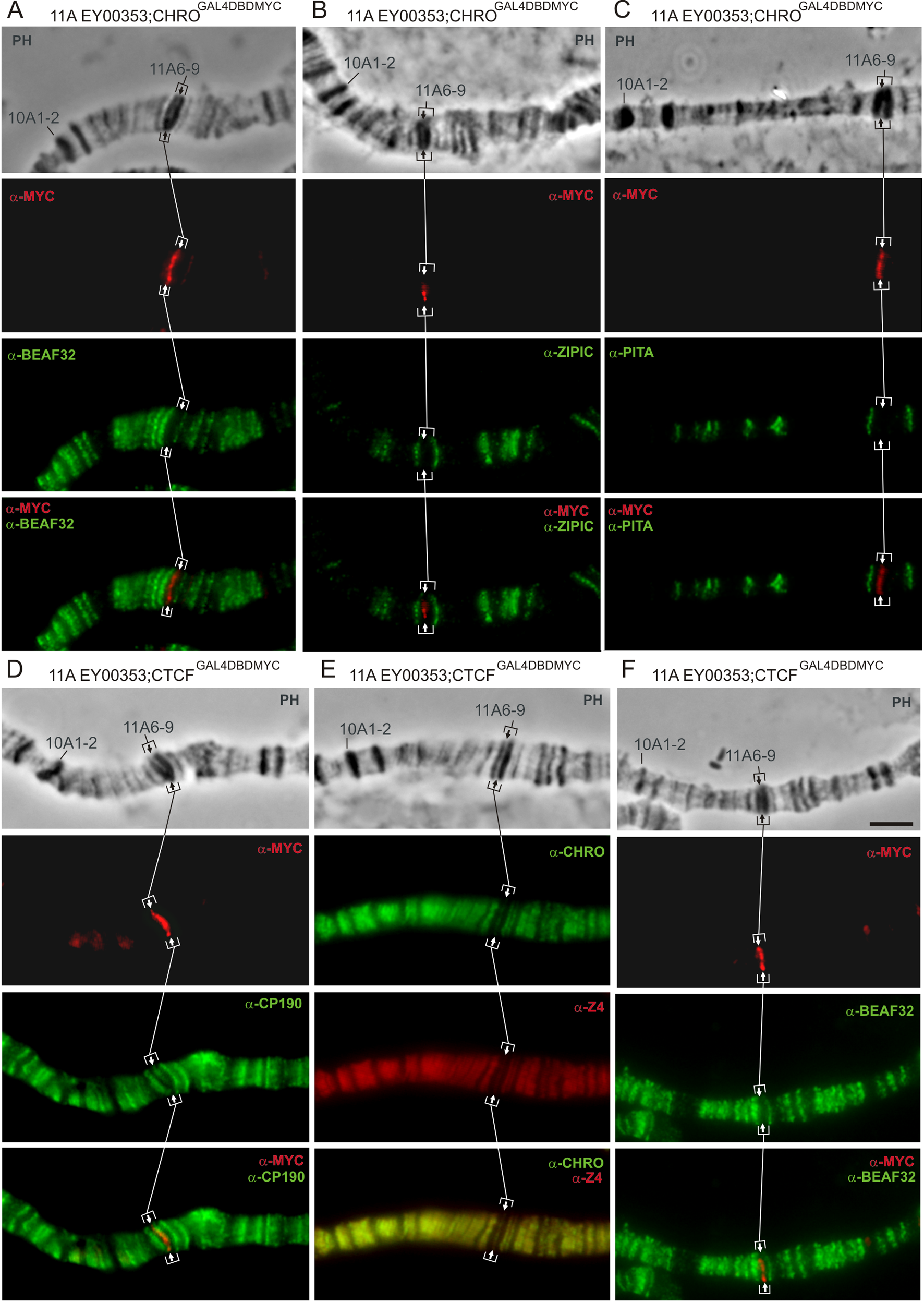

Supplement: S6 Fig — Tethering of dCTCFGAL4DBD induced binding of CP190, CHRO and Z4 proteins (D,E), but not BEAF32 protein (F). Each column (A-E) shows—phase contrast, immunostaining and overlay of immunostaining (from top to bottom, consequently). Arrows indicate the position of decompacted zone within 11A6-9 band. (TIF) [file pone.0192634.s006.tif]
