# Supplementary material for: Tethering of CHROMATOR and dCTCF proteins results in decompaction of condensed bands in the Drosophila melanogaster polytene chromosomes but does not affect their transcription and replication timing
Source: PLoS One. 2018 Apr 2;13(4):e0192634. doi: 10.1371/journal.pone.0192634 (PMC5880345; doi:10.1371/journal.pone.0192634)
Supplement: S1 Text — (DOC) [file pone.0192634.s007.doc]

**Supplementary TEXT**

To give information how the fluorescence signals label the chromosome regions here we describe consequent steps in labeling the chromosome sites. The set of antibodies and DNA sample were used. At the first panel (Suppl. Fig. 1) we show the chromosome under phase contrast (PH) to see how the chromosome structures look. The next pictures show splitting the 10A1-2 band upon the CHRO^GAL4DBD^ tethering, where A and C represent control, while B and D are experimental. At first α –MYC labelled in red demonstrate presence GAL4DBD. The next picture demonstrates localization of CHRO (green labeling) in all interbands of the chromosome, and the final picture is result of matches of α –MYC and CHRO^GAL4DBD^. On the Suppl.Figures 1C and D matches of CG15208 and phase contrast are shown.

Supplementary Fig. 2A and B demonstrate matches of phase contrast (PH), α –MYC and CHRO antibodies in EY01976 insertion in the distal part of 11A6-9, similar matches were prepared for insertion EY00353 insertion into the middle part of the 11A6-9 (C and D). On the Suppl. Figure 3A and B matches of the of phase contrast, α –MYC and CHRO antibodies in EY13417 insertion in the middle part of 59D1-4 are presented. The SupplementaryFig. 3C-E demonstrates matches after CTCF tethering (D) and HP1 (E). These data show that despite on tethering the fused protein GAL4DBD^HP1^ to the insertion EY00353 splitting the 11A6-9 does not occur (Suppl. Fig. 3E). Therefore the tethering any large protein will not lead to a disruption of the chromosome structure.

The SupplementaryFigures 4-6 demonstrate matches after staining of the newly formed interbands with antibodies against different proteins characteristic to active and inactive chromatin fragments. Chromatin marks of dense chromatin go away (Supplementary Fig.4 A-C), and active marks appear instead (D-F). Each column (A-F) shows: phase contrast (PH), immunostaining and overlay of immunostaining from upper to bottom row, consequently.

Immunodetection of insulator proteins in the decompacted regions present appeared within the bands 10A1-2 (Supplementary Figure 5A,B) and 11A6-9 (Supplementary Figure 5C-E). Insulator proteins CP190, CHRO and Z4 become associated with tethering of CHRO^GAL4DBD^ in the regions of UAS-10A (A,B) and EY00353 (C-D) insertions, but dCTCF protein was undetectable upon CHRO^GAL4DBD^ tethering in decompacted region (E). Each column on the SFig.5 (A-E) represents phase contrast (PH), immunostaining and overlay of immunostaining (from upper to bottom row, consequently).

Immunodetection of insulator proteins with tethering of CHRO^GAL4DBD^ and dCTCF^GAL4DBD^ in EY00353 insertions within 11A6-9 band are shown on the Supplementary Fig. 6. BEAF32, ZIPIC and PITA proteins were undetectable in decompacted regions after tethering of CHRO^GAL4DBD^ (A-C). Tethering of dCTCF^GAL4DBD^ induced binding of CP190, CHRO and Z4 proteins (D,E), but not BEAF32 protein (F). Each column (A-F) shows - phase contrast (PH), immunostaining and overlay of immunostaining (from top to bottom, consequently).

**S1 Fig**. **10А1-2 band splits upon CHRO^GAL4DBD^ tethering.** Immunostaining (A,B) and FISH (C, D) signals. Each column (A-D) shows - phase contrast (PH) for the fragment of the X chromosome (subdivision 10), immunostaining and overlay of immunostaining (from upper to bottom row, consequently). Left columns (A,C) show tethering GAL4DBD-MYC (control), right columns (B,D) show splitting the 10А1-2 band upon CHRO^GAL4DBD^ tethering. Black and white arrows point to the decondensed region, red arrow indicates the position of *CG15208* on the edge of band in control chromosomes (C) or in the distal fragment that has split from 10A1-2 upon tethering CHRO (D). CHRO is shown in green, MYC - red, FISH signal is red. Lines connect homologous regions of the chromosomes. Bar represents 5 μm.

**S2 Fig.** **11А6-9 band splits upon tethering of CHRO^GAL4DBD^ (A-D) into the regions of EY01976 (A,B) and EY00353 (C,D) insertions.** Each column (A-D) shows: phase contrast (PH), immunostaining and overlay of immunostaining (from upper to bottom row, consequently). Lines connect homologous regions of the chromosomes. Upper columns indicate EY01976 insertion (A - control, B – CHRO^GAL4DBD^ expression and splitting of the band 11А6-9 in its distal part), bottom columns indicate EY00353 insertion in the middle of the band (C – control; D – tethering of CHRO^GAL4DBD^ and splitting of the band 11А6-9 in its central part). The arrows point to the decompacted regions.

**S3 Fig**. **59D1-4 band splits in the heterozygotes for EY13417 insertion upon CHRO^GAL4DBD^ tethering (A,B) and splitting the 11A6-9 band in strain with EY00353 (C-E).** Control EY13417/+; GAL4DBD-MYC chromosomes (A); tethering of CHRO^GAL4DBD^ to one UAS-bearing homolog manifests as a partial splitting of the 59D1-4 band in its central part (B). The 11А6-9 band splits upon tethering dCTCF^GAL4DBD^ in EY00353 insertion in its central part (C,D). Control EY00353;GAL4DBDMYC, normal pattern binding of dCTCF protein in 10A1-2 – 11A region (C). Tethering of dCTCF^GAL4DBD^  and splitting band 11А6-9, the new binding site with dCTCF protein marks its decompacted part (D). Tethering HP1^GAL4DBD^ [67] in EY00353 insertion does not split 11А6-9 band (E). Each column (A-E) from upper to bottom row, consequently, shows - phase contrast (PH), immunostaining and overlay of immunostaining, consequently. Lines connect homologous regions of the chromosomes and the arrows point to the decondensed regions.

**S4 Fig.** **Epigenetic make-up of the newly formed interbands in the context of the 10А1-2 and 11А6-9 bands. Immunostaining signals for SUUR (A), H1 (B), H3 (C), H3K9ac (D), H3S10 (E), WDS (F).** The arrows denote the novel interbands formed at UAS sites in the bands 10A1-2 and 11А6-9, respectively. Chromatin marks dense chromatin go away (A-C), and active marks appear instead (D-F). Each column (A-E) shows - phase contrast (PH), immunostaining and overlay of immunostaining from upper to bottom row, consequently.

**S5 Fig. Immunodetection of insulator proteins in the decompacted regions present around and formed within the bands 10A1-2 (A,B) and 11A6-9 (C-E).** Insulator proteins CP190, CHRO and Z4 become associated with tethering of CHRO^GAL4DBD^ in the regions of UAS-10A (A,B) and EY00353 (C-D) insertions, but dCTCF protein was undetectable upon CHRO^GAL4DBD^ tethering in decompacted region (E). Each column (A-E) shows - phase contrast, immunostaining and overlay of immunostaining (from upper to bottom row, consequently). Arrows indicate the position of decompacted zone within 10A1-2 and 11A6-9 bands.

**S6 Fig. Immunodetection of insulator proteins with tethering of CHRO^GAL4DBD^ and dCTCF^GAL4DBD^ in EY00353 insertions within 11A6-9 band. BEAF32, ZIPIC and PITA proteins were undetectable in decompacted regions after tethering of CHRO^GAL4DBD^ (A-C).** Tethering of dCTCF^GAL4DBD^ induced binding of CP190, CHRO and Z4 proteins (D,E), but not BEAF32 protein (F). Each column (A-E) shows - phase contrast, immunostaining and overlay of immunostaining (from top to bottom, consequently). Arrows indicate the position of decompacted zone within 11A6-9 band
